# Testing and Refining the Ethical Framework for the Use of Horses in Sport

**DOI:** 10.3390/ani13111821

**Published:** 2023-05-31

**Authors:** Bluebell Brown, Jacqueline M. Cardwell, Kristien L. P. Verheyen, Madeleine L. H. Campbell

**Affiliations:** 1 Department of Pathobiology and Population Sciences, The Royal Veterinary College, Hawkshead Lane, Herts AL9 7TA, UK; 2 School of Veterinary Medicine and Science, University of Nottingham, Sutton Bonnington, Nottingham LE12 5RD, UK

**Keywords:** ethical framework, horse welfare, decision making, equestrian sports

## Abstract

**Simple Summary:**

Equestrian sport incorporates a number of different disciplines. To optimize equine welfare, a consistent method of ethical decision making is needed. The research presented in this paper was undertaken to test the practical applicability of a previously published theoretical ethical framework for the use of horses in sport. Stakeholders from a range of equestrian disciplines were invited to test the framework through using it both as individuals and in small groups to consider various relevant ethical dilemmas. Stakeholders fed back to the researchers their experiences of using the framework and suggestions for improving it, and the feedback was used to refine the framework across three rounds of engagement. Stakeholders found that the useability of the framework increased with each round, so that the finalised refined framework is a practical decision-making tool which can be used to optimize equine welfare through facilitating consistent, transparent decision making by a wide variety of stakeholders in equestrian sport.

**Abstract:**

In 2021, in response to an acknowledged need for universal, consistent ethics to guide decision making in the horse sport sector, Campbell published a theoretical ethical framework for the use of horses in competitive sport. The research reported here tested the applied usefulness of that theoretical ethical framework through stakeholder engagement in a three-round modified Delphi study and refined it to develop a practical decision-making tool which can be applied consistently across multiple equestrian disciplines. Stakeholders from a broad range of equestrian competitive disciplines participated in the research. Participants were required to apply the ethical framework to a pre-determined ethical dilemma, individually (Rounds 1 and 2) and within a group (Round 3), and at the end of each round to complete a questionnaire designed to gauge opinion and user experience. At the completion of each round of testing, the theoretical framework was refined based on stakeholder feedback. Results showed that participants perceived useability and application of the framework to generally increase with each round. Qualitative content analysis identified key concepts, including cognition (e.g., broadens/deepens thinking) and application (e.g., considers multiple angles from a variety of information sources, needs to be a short/simple process). Results suggested that the refined framework is beneficial for group decision making across a wide variety of ethical issues and equestrian competitive disciplines. The framework thus has the potential to improve equine welfare through facilitating consistent ethical decision making in which the interests of the horse are prioritized.

## 1. Introduction

Continuing advances in animal welfare science, legislation, and public awareness in the last two decades have culminated in an increased questioning of the ‘social licence’ for equestrianism to operate [1,2,3,4,5,6,7,8,9,10]. Campbell [9,11] has previously highlighted the lack of overarching ethics (a set of morals) within equestrian sport that would facilitate the ethical evaluation of proposed actions, potentially impacting equine welfare and social licence. To address this gap, Campbell [9] developed a question-based, nine-step, theoretical ethical framework for the use of horses in competitive sport. Rather than a prescriptive code, the framework is designed to enable a holistic, transparent, systematic, evidence-based evaluation of an ethical issue which facilitates contextual, consensual decision making, thereby contributing to the maintenance of the social licence of equestrianism to operate. This framework is a blend of ethical theories, incorporating utilitarianism (harm–benefit analysis), virtue ethics and deontology. Equine welfare is at the centre of the framework through its ‘central tenets’ which operate as the checks and balances of the framework, where any framework decision arrived at needs to meet the conditions of minimisation of negative equine welfare effects and maximisation of positive equine welfare effects and of identification and mitigation of avoidable, unnecessary risk.

The heightened interest in and need to consider ethical issues [12,13] are concurrent with the development of the framework. There is a theoretical framework, but we now need to test whether it works in practice, and to do that, we need stakeholder input, which could also encourage uptake [8,14]. Elite sport was chosen for testing because public interest and industry discourse around welfare impacts and the ethical context of using horses in sport has been the focus at the elite level.

Recently within academia, two general theoretical ethical frameworks for animal health decisions and animal use decisions have also been published [15,16]. These differ from Campbell’s framework, as they are not specific either to equids or to the use of animals in sport. Campbell’s framework, as tested here, incorporates a recognition both of the need for collective responsibility by all stakeholders and of stakeholders having their own starting points [9,17]. As this framework is a combination of ethical theories, it does not require stakeholders to ascribe to one particular belief system. In this way, contemplation or the actual use of this framework is less likely to be perceived as a freedom threat and elicit psychological reactance, which motivate the stakeholder to engage in the opposite behaviour to restore their freedom [18,19,20,21,22]. It is hoped that in this way, framework uptake may be considered by a wide variety of stakeholders, which promotes ethical decision making in horse sport across different sectors and roles of the equine industry.

The aim of the study reported in this paper was to test and further develop Campbell’s [9] theoretical ethical framework for horse sport into a practical tool that can be applied consistently for ethical decision making in equestrianism. The objectives were to (i) test the useability and application of this framework amongst a broad range of stakeholders in elite equestrian sport, across disciplines, and (ii) identify and implement framework refinements through stakeholder testing and feedback.

## 2. Materials and Methods

### 2.1. Study Sample

The panel of stakeholders was recruited through purposive heterogenous sampling. Inclusion criteria required participants to be involved with one or more of 11 specified competitive equestrian disciplines (dressage, show jumping, eventing, para-dressage, flat racing, jump racing, endurance, reining, carriage driving, para carriage driving, polo) whilst occupying one or more of 13 equine industry roles (competitor, trainer/coach, owner, groom, veterinarian, ambassador, regulator, sponsor, competition organiser, young rider (25 years old or under), animal welfare organisation, academia/education/research, breeder). A minimum of three participants per role was envisaged to allow for attrition during the project. Greater representation for the roles of competitor, trainer, veterinarian, regulator, and owner (four to six participants) was desired, as these are stakeholders who are routinely faced with decision making for sport horses. The projected total sample size was a maximum of 55 participants, which was felt to provide sufficient representation from different stakeholder roles across the equestrian disciplines whilst also being a small enough number to facilitate effective group working.

Potential participants were identified through personal contact networks of the researchers and the project review group, and they were provided with a brief overview of the purpose of the project. If the person indicated interest, a consent form alongside a project information sheet was emailed, to ensure that participants were able to freely provide informed consent to participate if they wished to do so [23]. On return of a signed consent form, the participant was formally enrolled into the study and assigned a unique reference number (URN) to anonymise data and to reduce the risk of researcher bias during data analysis [24,25].

This study included three Delphi rounds. Overall, 28 stakeholders participated in Round 1, 30 in Round 2, and 28 in Round 3. Thirty-eight participants initially consented to take part in the study. Seven withdrew during Round 1. At the end of Round 1, there was a second phase of participant recruitment to increase the sample size. Snowball sampling via enrolled participants and personal contact networks identified potential recruits. The enrolment process described above was repeated for new recruits. A further 13 participants were recruited. During Round 2, six participants withdrew.

In Round 1, respondents (28) occupied nine industry roles (competitor, trainer/coach, owner, vet, regulator, competition organiser, animal welfare organisation, academia/education/research). Competitor, owner, trainer/coach, vet, regulator, and academia categories were equally represented (14.3%; 4/28 in each category) followed by competition organisers (7.1%; 2/28), with breeders and animal welfare organisations least represented (3.6%; 1/28 in each category).

In Round 2, respondents (30) occupied 11 industry roles (competitor, trainer/coach, owner, vet, ambassador, regulator, competition organiser, animal welfare organisation, academia/education/research, course designer, breeder), with over 50% reporting involvement in more than one industry position (e.g., one participant identified as vet, regulator, academia/education/research, and another as owner and animal welfare organisation). There was similar representation within five categories: competitors (30%; 9/30), trainer/coach, owner, animal welfare organisation (26.7%; 8/30) and academia/education/research (23.3%; 7/30). This was followed by equal representation of breeders and competition organisers (17%; 5/30), vets and regulators (14%; 4/30), and ambassadors (7%; 2/30), with course designer as the least represented (1/30).

In Round 3, respondents (28) occupied 10 industry roles, with ‘ambassador’ no longer represented. Competitors and academia/education/research were equally represented (both 5/25; 20%), followed by animal welfare organisations (4/25; 16%); veterinarian, trainer/coach, regulator (3/25 in each category; 12%); and owner, breeder and competition organiser (2/25 in each category; 8%).

### 2.2. Data Collection

A three-round modified Delphi study was used to facilitate the iterative testing and refinement of the ethical framework. The study took place from December 2020 to June 2021. Within each round, participants were (a) required to apply the framework to answer an ethical question provided by the research team (e.g., ‘should there be an upper weight limit for polo players?’; Table S1) and (b) complete a questionnaire to gauge opinion, user experience, demographic variation and inform framework refinements (Document S1). The questionnaire comprised 17 open and closed questions, including six 7-point Likert items to inform the iterative process of the three rounds. The Likert items assessed three distinct concepts: framework useability, framework aids and framework application [26]. Within each round, participants were given three weeks to apply the framework and to return the questionnaire. A ‘finish later’ option was available to take account of time constraints and to provide time for reflection on the experience of using the framework [27,28].

After each round, the framework steps and guidance on how to use the framework were refined based on participant feedback. The refined framework was then tested in the subsequent round. Before the beginning of each subsequent round, participants who returned their applied framework and questionnaire received round-specific feedback. This summarised the key feedback provided as comments or responses to open-ended questions, along with the actions taken to refine the framework based on this feedback. Each round had unique elements, described below.

#### 2.2.1. Round 1

**Figure 1:**
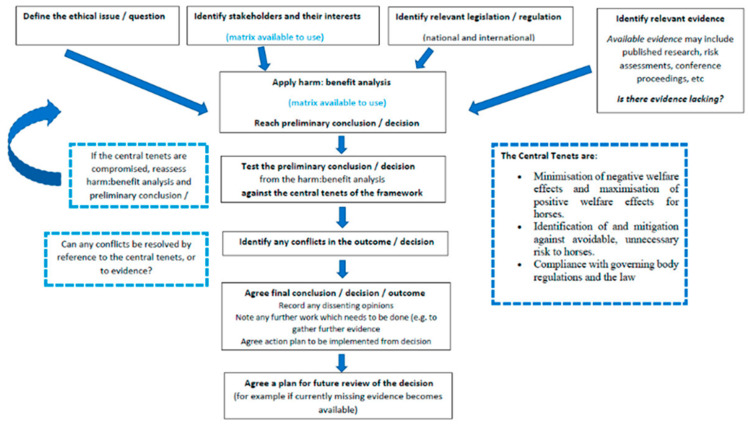
Diagrammatic step-by-step explanation of how to use the ethical framework [9].

Participants were asked to apply the ethical framework (Figure 1) and to complete the questionnaire individually online. A written step-by-step description of how to use the framework [9] was transferred to online surveys [29]. Participants received an automated invitation which included a personal link to access the questionnaire.

For Round 1, to facilitate framework familiarisation, all participants regardless of discipline involvement were asked the same general ethical question, ‘Should omeprazole be permitted for use in horses during competition?’ (Table S1).

A virtual information folder related to the pre-determined question was created to aid participants when completing the legislation/regulation step and evidence step of applying the framework during Round 1. This contained published peer-reviewed research and review articles [30,31,32,33,34,35], a European College of Equine Internal Medicine Consensus Statement [36], lay press articles [37,38,39,40], sporting body regulations [40,41,42,43,44,45,46,47,48,49] and omeprazole product characteristics [50,51]. A ‘how-to’ framework guide with a worked example (Document S2) and a ‘how to access and save the survey’ guide (Document S3) were also included in the information folder.

#### 2.2.2. Round 2

Following Round 1 feedback, and issues with reaccessing saved work in the online platform, participants were required to apply the ethical framework and complete the questionnaire individually within a Word document (Document S4) in Round 2. The document was emailed to each participant and returned via email. There was no information folder provided in this round; however, there was guidance at the beginning of the document on how to apply the framework and complete the questionnaire. This guidance was accompanied by a short video explaining the purpose of the research. The predetermined ethical question provided to participants in Round 2 was discipline-specific (Table S1). Participants were allocated to a specific discipline based on their questionnaire responses in Round 1 and were given the opportunity before the start of Round 2 to be allocated to a different discipline if they wished.

#### 2.2.3. Round 3

In Round 3, participants were required to apply the ethical framework to a predetermined discipline-specific question in pairs or groups of up to four people. Participants were assigned to pairs or groups based on discipline, industry role and performance/understanding in the previous rounds (as assessed from their responses and feedback by the researchers). This approach was taken to promote heterogeneous discussion and to support participants who had difficulty with some aspects of applying the framework in previous rounds. Prior to each pair or group session, participants were emailed the predetermined ethical question, the ethical framework document (Document S5), and a worked example of the Round 3 framework (Document S6). This helped to enable participants to gather sport rules/evidence sources prior to the group session, if they wished to do so.

One mixed discipline and ten discipline-specific group sessions took place between 20 April and 5 May 2021. Pair or group sessions were run online via Microsoft Teams for a duration of two hours. A member of the research team (BB) joined the session for the first 15–20 min, to introduce participants to each other, explain how the session would run and conduct an explanatory demonstration of the Round 3 framework, which also allowed for questions/clarification requests from participants. The group was asked to complete one framework Word document between them, and a volunteer notetaker within the group was chosen. Each member of the group was asked to complete an individual questionnaire independently and to return it via email within one week of the session. After this introductory segment, the researcher left the group to avoid the possibility of influencing interactions or responses but was contactable via the Microsoft Teams chat function for the remainder of the session to assist the group with any queries or difficulties. During the last 10 min of the session, the researcher re-joined the group to bring the session to a close. The designated notetaker returned the group framework Word document via email within one week (Document S5). This was to facilitate all members of the group checking the accuracy of notes from the session.

### 2.3. Data Analysis

At the end of each Round, the applied framework and questionnaire data were transferred to Microsoft Excel 21.02.

#### 2.3.1. Ethical Framework Application

Each returned ethical framework application document (Rounds 1 and 2—individual, Round 3—pair or group) was analysed using frequencies and percentages to determine whether the participant(s) attempted to complete each framework step and, if so, whether they completed the step correctly (as per given guidance). Percentages were compared between all rounds.

#### 2.3.2. Questionnaire

Responses to closed questions (demographics and Likert items) were transferred from Microsoft Excel 21.02 to SPSS .28 (Statistical Product and Service Solutions) and coded for statistical analysis.

The data were descriptively analysed. Median, interquartile range (IQR) and range were used to assess central tendency and data spread. Associations between each of the demographic variables (e.g., gender, income, Round 3 group) and Likert item responses were analysed using Fisher–Freeman–Halton exact tests. Prior to running the statistical tests, the 7-point Likert item responses were aggregated into disagree (strongly disagree, mostly disagree, somewhat disagree), neither agree nor disagree and agree responses (somewhat agree, mostly agree, strongly agree). Statistical significance was set at *p* < 0.05. Correlations between age and Likert item response were analysed using Spearman’s rho. Aggregated agree/disagree percentage for each Likert item was used to quantify level of agreement/disagreement among participants on the concepts of useability, framework aids and application. Differences in Likert item responses between Rounds 1 and 2 for cohort 1 (participants who began the study at Round 1 and completed Round 2) and between Rounds 2 and 3 (all participants) were analysed using Wilcoxon signed rank tests. Differences in the proportion of disagree/neither agree nor disagree/agree responses for the six Likert items between the participants who took part in Round 1 (*n* = 28) and participants who began study participation at Round 2 (*n* = 9) were analysed using Fisher–Freeman–Halton exact tests.

Qualitative data from the Likert items comment sections and open-ended questions were transferred from Microsoft Excel 21.02 to NVivo .21 after each round and were analysed using conventional content analysis (Hsieh and Shannon, 2005). An inductive approach was taken; there was no pre-existing coding frame, and data were analysed on a semantic level.

## 3. Results

### 3.1. Sample Size and Response Rate across the Rounds

Thirty-eight participants consented to take part in the study. During Round 1, seven participants withdrew, leaving a total sample of 31 participants. Of these, three did not return completed documents, giving a final response rate of 90.3% (28/31). Seven participants received telephone calls to answer queries about what they were being asked to do. Due to difficulties experienced with saving and reaccessing their applied framework in the online platform, two participants responded with soft copies via email.

Six participants withdrew during Round 2, due to time pressure, COVID-19, or the European equine herpesvirus outbreak, leaving a total of 37 participants. Of these, seven did not return completed documents, giving a final response rate of 81% (30/37). Overall, 28 stakeholders responded in both Round 1 and 2, and 9 in Round 2 only.

In Round 3, two participants did not reply to any correspondence or join group sessions, leaving a group session response rate of 93% (28/30). The questionnaire response rate was 89% (26/28); however, one file was corrupted, leaving analysable data from 25 of the 28 group participants. One group did not return their completed framework document.

### 3.2. Participant Demographics throughout the Rounds

Responses from participants showed that participant age ranged from 30 to 73 years in Round 1, with a median age of 57 (IQR 49 to 64.5). In Rounds 2 and 3, participant age ranged from 23 to 73 years, with medians of 59.5 (IQR 52–70.5) and 59 (IQR 48–69.5), respectively. Men and women were equally represented in Rounds 1 (14:14, 50%) and 2 (15:15, 50%), whereas within Round 3, there was a slight female majority (13:12; 52%).

In Round 1, 9 industry roles were represented, compared to 11 in Round 2, where over 50% reported involvement in more than one industry position. Six roles had equal representation in Round 1 (competitor, owner, trainer/coach, veterinarian, regulator, and academia, 14.3%; 4/28) compared to five ‘near equal’ representations in Round 2 (competitors (30%; 9/30), trainers, owner, animal welfare (26.7%; 8/30) and academia (23.3%; 7/30). An additional role (course designer) was identified in Round 2. Participants occupied 10 industry roles in Round 3, with ‘ambassador’ no longer represented. There was near equal representation across five categories: competitors, academia (both 5/25; 20%) and veterinarian, trainers, animal welfare organisations (4/25 each; 16%).

In all three rounds, the majority of participants received their main source of income from equestrian sport. Participants reported that they were involved with 11 disciplines; throughout the three rounds, involvement was highest amongst racing (50–72%) and the Olympic disciplines of eventing (47–52%), dressage (33–46%) and show jumping (23–40%).

### 3.3. Round 1 Results

#### 3.3.1. Ethical Framework Application

Of those who responded, 82% (23/28) attempted to apply the framework, and 100% (28/28) completed the questionnaire. In the regulation/legislation step of the framework, 64.3% (18/28) included sport rules and legislation. Seventy-one percent (20/28) of participants provided evidence and engaged with the harm–benefit analysis in some form. Forty-three percent (12/28) of participants applied the central tenets.

#### 3.3.2. Questionnaire

All the Likert items had a data range across five or more of the seven response options (Table 1). The median score for the statements ‘I understand how to complete each part of the framework’, ‘the framework steps enabled me to come to a conclusion on the specified issue’ and ‘I would use this framework to make decisions in the future’, was 4 (neither agree nor disagree), with aggregate agreement of 46%, 39% and 43%, respectively. The Likert statements ‘the worked example helped me understand how to use the framework’ and ‘the stakeholder matrix helped me to apply the harm–benefit analysis to the question’ both had a median of 5 (somewhat agree) and a data range of 7. The median for ‘I understand all the terms used in the framework’ was 6 (mostly agree).

There was a statistically significant association between industry role as the main income source and increased agreement with Likert statements ‘The stakeholder matrix helped me to apply the harm–benefit analysis to the question’ (*p* = 0.02) and ‘The framework steps enabled me to come to a conclusion on the specified issue’ (*p* = 0.05). Participants who indicated their role was not their main income source were mostly owners (4/6), and owners predominantly disagreed with these two statements.

There were statistically significant negative correlations between age and the Likert statements ‘the stakeholder matrix helped me to apply the harm–benefit analysis to the question’ (r_s_ = −0.410, *p* = 0.03) and ‘the framework steps enabled me to come to a conclusion on the specified issue’ (r_s_ = −0.403, *p* = 0.03).

Response rate to the open-ended question ‘what do you like about the framework?’ was 100% (28/28). The responses mainly centred around cognition; participants considered the framework to be logical and structured, providing prompts to broaden thinking and to help reaffirm/challenge their opinions on the topic. Participants also commented on ‘evidence’, because the framework required the use of a variety of evidence sources; this ensured a well-rounded conclusion. For five of the participants, their response to the question ‘what do you like about the framework?’ was “not much/very little”. The question ‘what do you think could be improved?’ had a response rate of 96% (27/28), with responses relating mostly to application and interpretation. Nine participants felt that the framework needed to be simpler, shorter, and less academic; they considered it currently too time consuming with too much information (information folder). One participant commented: “Not sure it’s for people like me who is a practical person, I am not an academic”. The key concepts generated from the Likert items centred around interpretation and application but also featured cognition (Table 2).

#### 3.3.3. Round 1 Modifications

**Figure 2:**
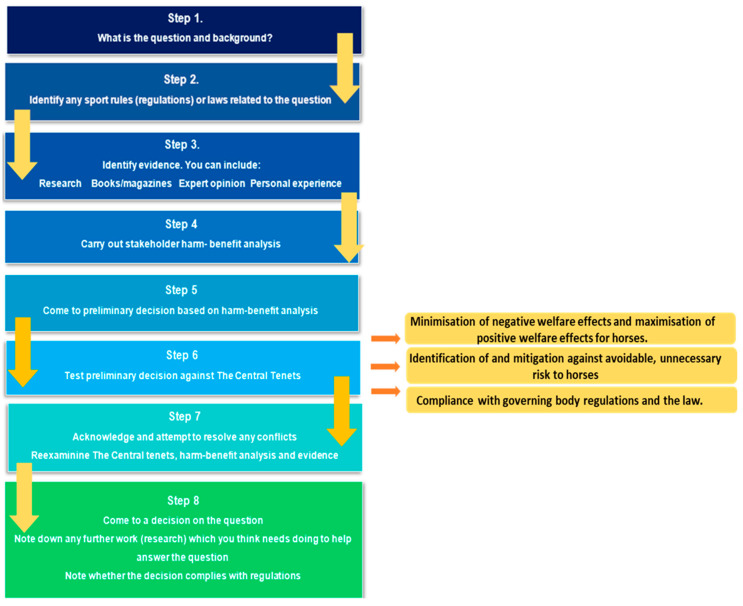
Diagrammatic step-by-step guidance of how to use the modified ethical framework used in Round 2.

Based on Round 1 results, actions were taken to address perceived framework limitations. An explanatory video was included to help participants understand the purpose of the framework and the testing process. Increased guidance was provided within the framework rather than as a separate document, for ease of access. In the written step-by-step framework guidance, information was provided on different types of literature to include where to search, and what information was needed to apply the framework correctly. The sport rules/legislation step included online links to discipline-specific sporting body rules and regulations. Similar framework steps were combined to simplify and shorten the process, alongside rewording the framework content into lay terms to make the process more accessible to non-academics. Stakeholder roles were explicitly defined. An explanation of the purpose of the stakeholder matrix was included in the written step-by-step framework guidance, in conjunction with clarification on how to conduct the harm–benefit analysis. A different worked example using an alternative question, and the modified Round 2 (Figure 2) framework steps and guidance was included.

### 3.4. Round 2

#### 3.4.1. Ethical Framework Application

Of those that responded, 100% (30/30) attempted to apply the framework and completed the questionnaire, compared to 82% in Round 1. Two participants (6.7%) entered irrelevant information for some steps, indicating misunderstanding. One participant did not apply any of the framework steps to arrive at their decision. Eighty-seven percent (26/30) applied the central tenets to decisions as intended. Three participants (10%) had software-related problems with viewing the framework, which affected how they answered. In the evidence step of the framework, 83% (25/30) included some form of ‘evidence’. Sixty-four per cent (16/25) included research evidence, with 69% (11/16) citing more than one research source. Sixty-eight per cent (17/25) included book/report/magazine evidence, with 87% (13/17) citing more than one source. Fifty-two per cent (13/25) included personal experience/opinion as a form of evidence, 15% (2/13) referred to plural experiences/opinions. Ninety-three per cent (28/30) engaged with the harm–benefit analysis in some form.

#### 3.4.2. Questionnaire

As in Round 1, Likert items had a data range across five or more of the seven response options (Table 3). There was an increase in the median score (e.g., 4 to 5, 5 to 6) for all statements, except for ‘the stakeholder matrix helped me to apply the harm–benefit analysis to the question’, which remained unchanged at 5 (somewhat agree). The largest increase in aggregate agreement was for ‘the framework steps enabled me to come to a conclusion on the specified issue’ (31%), followed by a 27% agreement increase in ‘I understood how to complete each part of the framework’, with one participant commenting: “As this was a subject I had more knowledge of it was much easier to complete than the initial round”.

For cohort 1 (21 participants who took part in both Round 1 and Round 2), there was a significant increase in agree responses for Round 2 compared to Round 1 for the Likert statements ‘I understood how to complete each part of the framework’ (Z = −2.213, *p* = 0.03) and ‘the framework steps enabled me to come to a conclusion on the specified issue’ (Z = −2.173; *p* = 0.03).

When comparing Likert item responses for the original framework in Round 1 (28 participants who took part in Round 1) with those for the revised framework in Round 2 (cohort 2: 9 participants who began the study at Round 2), there was a statistically significant difference in response for the Likert item ‘the stakeholder matrix helped me to apply the harm–benefit analysis to the question’ (*p* = 0.02), which had decreased disagreement and agreement and increased neither agree nor disagree. Descriptively, cohort 2 had an increased median (by one Likert score, e.g., neither agree nor disagree increased to somewhat agree) for the statements ‘I understood how to complete each part of the framework’, ‘the worked example helped me understand how to use the framework’ and ‘the framework steps enabled me to come to a conclusion on the specified issue’. Medians for ‘I understood all the terms used in the framework’ and ‘I would use this framework to make decisions in the future’ were the same, while ‘the stakeholder matrix helped me to apply the harm–benefit analysis to the question’ decreased (somewhat agree to neither agree nor disagree).

Comment response rate to the open-ended question, ‘what do you like about the framework?’ was 93% (28/30). The two key concepts identified were cognition and application. Participants found that the framework focused/deepened/broadened thinking related to ethics and welfare, and they liked the thorough, rational, and structured process the framework provided for decision making. Participants liked the consideration of a variety of stakeholders and the combination of science and practical elements within the framework decision-making process. Participants found the worked example very helpful.

The open-ended question ‘What do you think could be improved?’ had a response rate of 83% (25/28). The core issue was application. Participants reported that the framework needed to be made simpler, shorter, and easier to navigate.

Responses for the comment section of the Likert items also centred around application, interpretation, and cognition (Table 4).

#### 3.4.3. Round 2 Modifications

**Figure 3:**
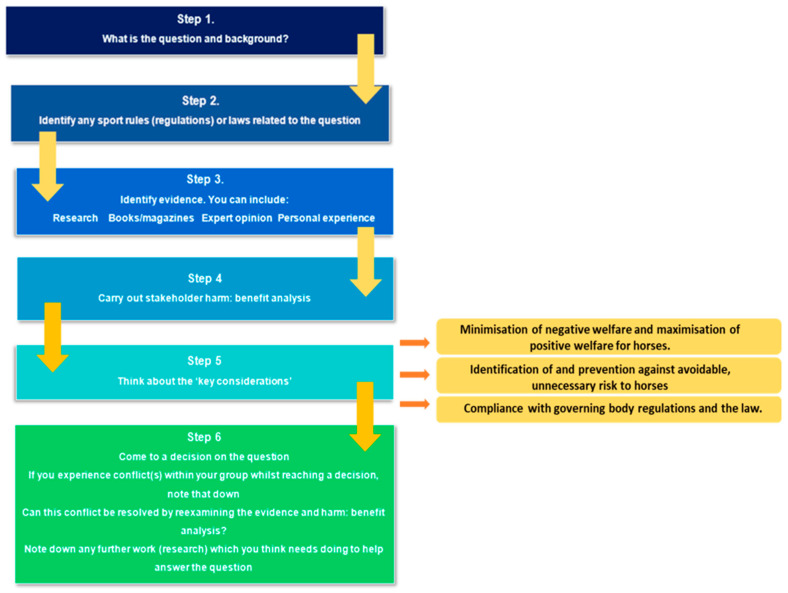
Diagrammatic step-by-step guidance of how to use the modified ethical framework used in Round 3.

Based on Round 2 results, specific actions were undertaken to address framework limitations as perceived by participants. Several framework steps were combined to further simplify and shorten the process, alongside rewording of some content (Figure 3). On the basis of participant suggestion, possible stakeholders were all included within the harm–benefit analysis table. The worked example was removed from an appendix and was provided as a stand-alone document, to decrease document scrolling time.

### 3.5. Round 3

#### 3.5.1. Ethical Framework Application

As with Round 2, of those that responded, 100% attempted some framework steps. One group (compared to two participants in Round 2) entered irrelevant information for the latter steps, pointing to misunderstanding. Eighty per cent of groups (8/10) included some form of ‘evidence’ compared to 83.3% (25/30) in Round 2, with time stated as a factor for one group who did not include evidence. Frequency of types of evidence used were consistent between Rounds 2 and 3. All groups (10/10) identified sport rules and engaged with the stakeholder matrix compared to 77% and 93%, respectively, in Round 2. All groups’ decisions (100%) complied with key considerations 1 and 2 (1: minimisation of negative welfare and maximisation of positive welfare for horses; 2: identification and prevention against avoidable unnecessary risk to horses).

#### 3.5.2. Questionnaire

**Figure 4:**
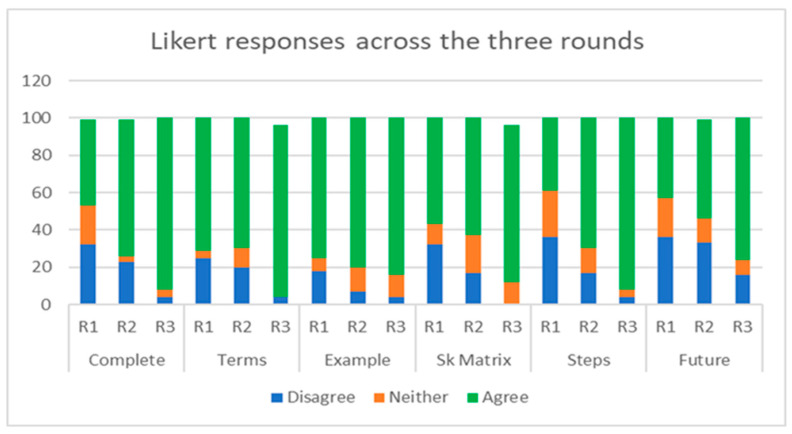
Participant Likert response % (disagree/neither agree nor disagree/agree) across the three rounds (Complete: ‘I understood how to complete each part of the framework’. Terms: ‘I understood all the terms used in the framework’. Example: ‘The worked example helped me understand how to use the framework’. Sk Matrix: ‘The stakeholder matrix helped me to apply the harm–benefit analysis to the question’. Steps: ‘The framework steps enabled me to come to a conclusion on the specified issue’. Future: ‘I would use this framework to make decisions in the future’. R1: Round 1; R2: Round 2; R3: Round 3).

In Round 3, the median score for all Likert questions was 6 (mostly agree). The statements ‘I understood how to complete each part of the framework’ (Z = −2.403. *p* = 0.01), ‘I understood all the terms used in the framework’ (Z = −2.093, *p* = 0.04), and ‘the framework steps enabled me to come to a conclusion on the specified issue’ (Z = −2.066, *p* = 0.01), all had aggregate agreement of 92%, which was a statistically significant increase compared to Round 2, with one person disagreeing with these statements (4%) (Table 5 and Figure 4). The statements ‘the worked example helped me understand how to use the framework’ and ‘the stakeholder matrix helped me apply the harm–benefit analysis to the question’ both had an aggregate agreement of 84%. Both statements had a decreased range of 4, with no disagreement scores. There was also a statistically significant increase in agreement for the statement ‘I would use this framework to make decisions in the future’ (Z = −2.066, *p* = 0.04). The majority of disagree scores in Round 3 (71%) were from one group.

The comment response rate to both open-ended questions was 92%. The key concepts for the question ‘What do you like about the framework?’ were again cognition and application, largely reflecting responses from the Likert items comment section (Table 6). Once again, participants liked the structured and step-based framework that can be applied to answer a question, with one participant reporting that it created “forced reflection and awareness”. Some participants stated that they could now understand the framework, finding it “a lot simpler than previous rounds” while one participant commented “the introduction session was crucial”. The logical and holistic nature of the framework enabling in-depth ‘outside the box’ analysis was again highlighted. Participants found the group format positive, liking the varied range of experience and roles within the group, enabling good discussion. Some participants reiterated that they liked the use of a variety of evidence sources, including personal experience, while conversely, one participant again emphasised the importance of “having all the facts, not just those in a report”. One participant’s response to the question ‘What do you like about the framework?’ was “not much”.

The question ‘What do you think could be improved?’ had key issues of application and interpretation. Some participants reported that it would be beneficial to alter the framework to a more streamlined online format that is easier to navigate than a Word document. Participants again highlighted the need for a highly specific framework question and instruction on applying the harm–benefit analysis. Participant suggestions included writing the framework question on each of the framework steps to keep the discussion on track, implementation of strategies to prevent social bias in groups, and changing the stakeholder matrix back to a list from which to choose. However, in contrast to other disciplines, racing participants stated the need to widen the stakeholders included in the harm–benefit analysis. In terms of interpretation, participants suggested the provision of more information on the purpose of the framework and clarity on stakeholder roles. The need to include all forms of evidence, not just those in a report, was again stated.

## 4. Discussion

The research presented in this paper aimed to test and refine Campbell’s [9] ethical framework from a theoretical concept to a tool which can be applied practically by stakeholders. The iterative process of the modified Delphi technique that was used facilitated the ‘testing’ by stakeholders of the framework for horse sport across three rounds, with refinements to the framework being made between rounds. To investigate the theoretical framework’s usability, it was fundamental to assess participants understanding, defined as ‘determining the meaning of instructional messages, including oral, written, and graphic communication’ [52]. Previous work has demonstrated that cognitive processing operates within a hierarchy, where attaining lower-order cognitive skills is a prerequisite for higher-order thinking; in this case, to be able to apply the framework to a question, analyse the evidence and evaluate the decision, you first have to understand how to complete the framework steps [52,53,54,55,56]. There was evidence of increased understanding from Round 1 to 2 as well as increased application of all framework steps. These changes in participant responses may be related to the move from generic to discipline specific questions, as well as the testing or practicing effect [57,58,59]. Learning (the acquisition of knowledge) occurs through active processing, where prior experience and incoming information integrate to create a mental model [54,60,61,62] and testing or practice requires active retrieval of information, compared to just encoding with repetitive reading [28,54,58,59,63,64,65]. Multiple studies have empirically validated the test effect on the transfer and retention of knowledge and student performance [57,58,66], especially with repeated ‘no-stakes testing’ (there is no positive or negative consequence as a result of the test) [54,59]. While some framework steps were reordered in Round 2, the fundamental components of evidence gathering, applying a harm–benefit analysis, and the central tenets remained the same. Therefore, Round 1 and 2 essentially acted as test/practice activities for participants who started at the beginning of the study. However, when comparing Likert scores of ‘first-time participants’ between the original framework in Round 1 and the revised framework in Round 2, there was increased agreement for those who began the study at Round 2 using the revised framework. This suggests that framework modifications and the provision of a new worked example were beneficial. The main Round 1 modifications centred around increased guidance for steps, altered wording of the written step-by-step framework guidance and a new worked example, which may have increased understanding of the framework and its application in Round 2.

The largest increase in participant agreement on the perceived useability and application of the framework and framework aids occurred after Round 3 (group use of the framework). Unlike a classical consensus-building Delphi study, the primary aim of this modified Delphi was not to reach a consensus or to use consensus as the determinant for ending one round and beginning another. Nevertheless, Round 3 reached consensus for all six Likert items; 75% agreement has been found as the median threshold to define consensus in Delphi studies [67].

As with the positive changes in participant opinion after Round 2, participants’ increased agreement in Round 3 may be related to the framework modifications and a testing or practicing effect (see above). Based on previous research and participant comments, collaborative and cooperative learning (which can occur during group work) is also likely to have had an impact on participant opinions, suggesting that the framework is well suited to group use. Collaborative learning is the culmination of individual or unique knowledge, contributing to the task that is carried out by the group as a whole, and cooperative learning occurs when individuals work together toward a shared learning goal [59,68]. Participants reported that having a variety of people with different skills, knowledge and experience within the group, and the resulting discussions, were beneficial to the decision-making process. However, previous research findings suggest that group work and decision quality is multifactorial rather than linear, where decision demonstrability, member ability, task instruction, team size and group dynamics converge to affect group experience and decision quality [69,70,71,72]. Social bias (either implicit or explicit) emerged within one group in Round 3, which affected a participant’s contribution to, and experience of, the decision-making process. This highlights that further modifications (to the process) are required to ensure that all participants’ views are considered equal.

It is anticipated by the researchers that the framework is most likely to be used by equestrian sport in group decision-making scenarios, and the framework was designed with that in mind. It was thus encouraging to find that a large majority of participants agreed that they would use the framework within groups in the future. Four participants disagreed, two of whom belonged to the same group. However, the comments indicated that disagreement was likely based on misunderstanding the aim of the ethical framework, misinterpretation of evidence sources that can be included within the framework, and the simulated group sessions, which took place in one sitting and do not reflect the real-world scenario.

Key participant concepts identified in the open-ended questions generally remained constant throughout the three rounds. These concepts centred around the structured decision-making process, the consideration of matters from different perspectives and the need for a simple process. This could be related to the kind of decision making that is required for framework use: critical decisions require increased endeavour and higher-order cognitive skills, which is a more complex process compared to routine or daily decision making [73].

These results suggest that the framework is useful for group decision making on a wide variety of ethical questions across equine competitive disciplines but will require further modifications arising from Round 3 testing. This work is underway and ongoing. It includes the development of an alternative format to a Word document, that is available online, as previous participants highlighted the importance of keeping the framework process simple, accessible, efficient, and easy to understand. Participants also requested shortened explanations after Round 3, as with increasing practice, they did not require extensive guidance. A solution to this is developing the framework into a multi-layered, online format, where core guidance is central, but there is an option to easily access more extensive guidance while becoming familiar with the framework process. During Round 3, participants had a researcher-led introductory discussion segment in their groups which was deemed as a facilitator for using the framework. In conjunction with the multi-layered online format, a tutorial will be developed with the aim of enabling ease of use and familiarising stakeholders with the function and application of the framework. Both the alternative format and tutorial design will be theoretically and empirically informed, to promote the transfer and retention of the key information needed to apply the framework correctly, while keeping the process simple. Other framework refinements centre around the harm–benefit analysis, including clarifying stakeholders that can be applied during the step process and how to apply the harm–benefit analysis. This will be facilitated using an integrated glossary and the multi-layered nature of the framework instruction.

### Limitations

As the framework had been developed for use by a variety of stakeholders across a broad range of disciplines, it was important to investigate if there was any variation in participant Likert responses by demographic variables, e.g., age, group, industry role, industry role as the main source of income, and Round 3 group. However, it was not possible to include industry role and self-allocated discipline in this analysis because many participants had multiple roles in multiple disciplines. Analysis was further limited by the somewhat uneven distribution of participants across disciplines and variation across rounds (as described in Materials and Methods). Nonetheless, the qualitative opinions highlighted a difference between racing and the other eight disciplines. Several participants allocated to racing raised the importance (in Round 2 and 3) of conducting an extensive harm–benefit analysis with all relevant stakeholders included in the stakeholder matrix. In contrast, other disciplines commented that the stakeholder matrix was too extensive, with the suggestion of reducing the stakeholder list. This discrepancy may be related to the perceived importance of securing a level of social licence; as racing is the second most popular spectator sport in the United Kingdom, racing and racing-related injuries have a high profile within the public domain and animal rights organisations [74,75].

During Round 3, engagement was negatively impacted by a single decision-making session per group with a length of two hours, with several participants reporting that there was insufficient time. A Delphi study requires substantial commitment from participants to engage with each round [76,77]. As participants were already completing three separate rounds, it would have been unfeasible to expect participants to conduct Round 3 over several sessions.

The group round, which by its nature breaks usual anonymity of a Delphi consultation and creates intragroup dynamics (positive or negative), could be regarded as a study limitation. However, in a real-world scenario, stakeholders making decisions on regulatory issues within the competitive equine industry will be doing so collectively [78], and it was therefore necessary to test this scenario. Questionnaires in all three rounds were completed individually after the decision-making process; intragroup dynamics did not therefore impact questionnaire responses or bias these results.

Finally, this study looked at participant opinion on a surface level. Cognitive and social psychology theory and research have demonstrated that both implicit and explicit biases can impact critical thinking but also function as sociocognitive facilitators and challenges, which influence behaviour [56,79,80,81,82,83,84] and thus uptake and correct use of framework. This needs to be investigated further.

## 5. Conclusions

All stakeholders in equestrianism have a shared and collective responsibility for ensuring that ethical decision making and equine welfare are at the heart of equestrian sport. To facilitate the acceptance of such responsibility and effective decision making, a holistic, transparent, systematic, evidence-based method of evaluating an ethical issue is required. The adoption of a standard method across equestrian disciplines makes it easier to explain transparent, evidence-based decision making to the wider public [9]. The results reported here demonstrate that the ethical framework for the use of horses in sport, as revised during the course of this research and particularly when used on a group basis, is an understandable, useable, and effective tool that can be used by a wide variety of equine industry stakeholders to make ethical decisions in a range of disciplines.

## Figures and Tables

**Table 1:** Distribution of responses from 28 participants in Round 1 of a Delphi study to test the usability and application of an ethical framework for decision making in horse sport. Aggregate disagree and agree percentages are highlighted in dark grey. Neither agree nor disagree score (N agree/disagree) are highlighted in light grey. Median percentage (the middle value) is highlighted in black.

Score	Likert Statement (% Responses)					
	I understood how to complete each part of the framework	I understood all the terms used in the framework	The worked example helped me understand how to use the framework	The stakeholder matrix helped me to apply the harm–benefit analysis to the question	The framework steps enabled me to come to a conclusion on the specified issue	I would use this framework to make decisions in the future
Strongly Disagree	0	0	3.6	10.7	7.1	21.4
Mostly Disagree	14.3	7.1	7.1	0	10.7	10.7
Somewhat Disagree	17.9	17.9	7.1	21.4	17.9	3.6
Aggregate Disagree	32.1	25	17.9	32.1	35.7	35.7
N agree/disagree	21.4	3.6	7.1	10.7	25	21.4
Somewhat agree	32.1	17.9	28.6	28.6	17.9	28.6
Mostly Agree	14.3	46.4	28.6	17.9	17.9	14.3
Strongly Agree	0	7.1	17.9	10.7	3.6	0
Aggregate Agree	46.4	71.4	75	57.1	39.3	42.9

**Table 2:** Round 1 key concepts from questionnaire Likert item comments. (Likert item 1: ‘I understood how to complete each part of the framework’. Likert item 2: I understood all the terms used in the framework’. Likert item 3: ‘The worked example helped me understand how to use the framework’. Likert item 4: ‘The stakeholder matrix helped me to apply the harm–benefit analysis to the question’. Likert item 5: ‘The framework steps enabled me to come to a conclusion on the specified issue’. Likert item 6: ‘I would use this framework to make decisions in the future’).

Likert Item	Response %	Concept Title	Concept Description
**1**	85	**Interpretation**	Unsure of literature searching for framework steps, length of answer required, and whether completing framework correctly.
		**Application**	Framework a long process that was complicated and wordy.
**2**	43	**Interpretation**	Difficulty understanding framework steps. Difficulty separating out some of the framework steps. Several participants found the framework terms clear.
		**Application**	Need for clarity on stakeholder roles for use in the stakeholder matrix was highlighted. Difficulty applying steps within the context of the pre-determined ethical question.
**3**	43	**Application**	Example helpful but unsure of how to apply the example to their given question. One participant commented that there was little personal experience evidence included within the example.
**4**	39	**Application**	Stakeholder matrix reported as helpful and a logical process. Participant indicated did not understand the purpose or application of the matrix. Clarity on how to apply the harm–benefit analysis requested.
**5**	50	**Cognition**	Framework did not enable a conclusion per se but did provide prompts to broaden thinking and helped to reaffirm/question an individual’s position.
		**Application**	The need to simplify the process was raised. Clarity on information sources for framework steps needed.
**6**	50	**Application**	Future framework use dependent on what the decision would be for; framework process too long, time-consuming and complicated. A participant with the industry role of competitor queried the use of the framework for hands-on stakeholders.
		**Cognition**	A logical method that broadened thinking

**Table 3:** Distribution of responses from 30 participants in Round 2 of a Delphi study to test the usability and application of an ethical framework for decision-making in horse sport. Aggregate disagree and agree percentages are highlighted in dark grey. Neither agree nor disagree score (N agree/disagree) is highlighted in light grey. Median percentage (the middle value) is highlighted in black.

Score	Likert Statement (% Responses)					
	I understood how to complete each part of the framework	I understood all the terms used in the framework	The worked example helped me understand how to use the framework	The stakeholder matrix helped me to apply the harm–benefit analysis to the question	The framework steps enabled me to come to a conclusion on the specified issue	I would use this framework to make decisions in the future
Strongly Disagree	6.7	3.3	0	0	0	3.3
Mostly Disagree	3.3	6.7	0	3.3	10	20
Somewhat Disagree	13.3	10	6.7	13.3	6.7	10
Aggregate Disagree	23.3	20	6.7	16.7	16.7	33.3
N agree/disagree	3.3	10	13.3	20	13.3	13.3
Somewhat agree	30	13.3	16.7	16.7	40	20
Mostly Agree	40	43.3	36.7	33.3	23.3	26.7
Strongly Agree	3.3	13.3	26.7	13.3	6.7	6.7
Aggregate Agree	73.3	70	80	63.3	70	53.4

**Table 4:** Round 2 key concepts generated from questionnaire Likert item comments (Likert item 1: ‘I understood how to complete each part of the framework’. Likert item 2: I understood all the terms used in the framework’. Likert item 3: ‘The worked example helped me understand how to use the framework’. Likert item 4: ‘The stakeholder matrix helped me to apply the harm–benefit analysis to the question’. Likert item 5: ‘The framework steps enabled me to come to a conclusion on the specified issue’. Likert item 6: ‘I would use this framework to make decisions in the future’).

Likert Item	Response %	Concept Title	Concept Description
**1**	37	**Interpretation**	Difficulty understanding ‘conflicts box’, the central tenets and some language/terminology. Found Round 2 intuitive, easier, well-written instructions.
**2**	23	**Interpretation**	Difficulty understanding the central tenets. Participant needed to look up ‘utilitarianism’.
		**Application**	Very long process, lots of time spent document scrolling.
**3**	33	**Application**	Very useful; core reference when completing Round 2. One participant had IT difficulties and could not refer to example; one participant forgot there was an example; one participant found an example at the end.
**4**	33	**Application**	Participant highlighted the importance of including all stakeholders that may be impacted in the harm–benefit analysis. Helpful, and it was good to have examples in Round 2. Participant suggestion of altering the matrix tabulation. Difficulty summarising and/or ‘weighting’ different stakeholders. Participant raised importance of including personal experience within competition environment as evidence (as this impacted their final decision).
		**Cognition**	Harm–benefit analysis prompted areas of thinking which would not have been considered if this step was not included.
**5**	40	**Cognition**	Framework broadens thinking, where conclusion is based on structured thought process that identifies and collates evidence, rather than relying on opinion.
		**Interpretation**	Participant concerned their final conclusion not ‘strong’ enough; participant queries if framework makes decision process ‘too standardised’. Participant felt they would benefit from further explanations of framework steps.
		**Application**	Need for including personal experience again highlighted.
**6**	47	**Application**	Facilitates structured arguments with pros and cons, could be used in many areas of equine industry; good concept but process too long; participant would not use it quite as constructed but would use some elements of it.
		**Interpretation**	Framework helpful when reviewing sport rules, not just for game but for welfare; participant not convinced of the need for a formal framework.

**Table 5:** Distribution of responses from 25 participants in Round 3 of a Delphi study to test the usability and application of an ethical framework for decision making in horse sport (Likert item 1: ‘I understood how to complete each part of the framework’. Likert item 2: ‘I understood all the terms used in the framework’. Likert item 3: ‘The worked example helped me understand how to use the framework’. Likert item 4: ‘The stakeholder matrix helped me to apply the harm–benefit analysis to the question’. Likert item 5: ‘The framework steps enabled me to come to a conclusion on the specified issue’. Likert item 6: ‘I would use this framework to make decisions in the future’). Dark grey = aggregate agree and disagree. Light grey = N agree/disagree. Black = median.

Score	Likert Statement (% Responses)					
	I understood how to complete each part of the framework	I understood all the terms used in the framework	The worked example helped me understand how to use the framework	The stakeholder matrix helped me to apply the harm–benefit analysis to the question	The framework steps enabled me to come to a conclusion on the specified issue	I would use this framework to make decisions in the future
Strongly Disagree	0	0	0	0	0	4
Mostly Disagree	4	0	0	0	4	8
Somewhat Disagree	0	4	0	0	0	4
Aggregate Disagree	4	4	0	0	4	16
N agree/disagree	4	0	12	12	4	8
Somewhat agree	12	0	8	28	12	20
Mostly Agree	60	64	48	40	56	32
Strongly Agree	20	28	28	16	24	24
Aggregate Agree	92	92	84	84	92	76

**Table 6:** Round 3 key concepts generated from questionnaire Likert Item comments (Likert item 1: ‘I understood how to complete each part of the framework’. Likert item 2: I understood all the terms used in the framework’. Likert item 3: ‘The worked example helped me understand how to use the framework’. Likert item 4: ‘The stakeholder matrix helped me to apply the harm–benefit analysis to the question’. Likert item 5: ‘The framework steps enabled me to come to a conclusion on the specified issue’. Likert item 6: ‘I would use this framework to make decisions in the future’).

Likert Item	Response %	Concept Title	Concept Description
**1**	32	**Application**	Participants reported that it would be beneficial if the ethical question had more specific phrasing. How to apply the harm–benefit analysis was raised, as was the need for participant guidance when there was a lack of available evidence. Participants commented on the discussion within the group round and that the process became easier with familiarity. The usefulness of the example was also mentioned, as was the helpfulness of having all stakeholders within the matrix. One participant commented on the inclusion of research, complicated terminology, and a lack of inclusion of hands-on experience.
**2**	32	**Application**	Some participants reported that it was now much easier and that they felt that the adaptations had worked; one participant struggled; one found that the terms were too academic. How to apply the harm–benefit analysis was raised.
		**Interpretation**	The ambiguity of stakeholder roles was raised.
**3**	36	**Application**	Some participants found the worked example ‘invaluable’, while it was very helpful, they did not need to refer to it in Round 3. One participant stated that a more ‘in-depth’ work up would be beneficial. The use of research but lack of other ‘knowledge’ in the example was again raised.
**4**	44	**Cognition**	Participants reported inclusion of the harm–benefit analysis stimulated discussion and enabled consideration from lots on angles.
		**Application**	The issue of how to apply the harm–benefit analysis was raised again. Racing participants felt that the stakeholder list could be more extensive, while participants from other disciplines found the list long, with the risk of burnout.
**5**	40	**Cognition**	Framework process worked well in groups and progressed through the framework steps logically, highlighting different aspects of an issue even when familiar with the topic; with a real-life question, the framework would be helpful.
		**Application**	While framework steps were good, more time was needed within the group sessions to work through them. Specificity of the question was raised again, while one participant felt that their group question was incorrectly framed. Difficulty in coming to a ‘definite’ conclusion was raised. Not including information/knowledge that is not documented on paper was raised again, as this may lead to a conclusion without all the facts and information. One participant reported that a social bias/hierarchy within their group emerged, based on experience and role within the industry, which influenced the discussion and decision-making process.
**6**	44	**Cognition**	Process was really helpful in groups/pairs, structuring the discussion, allowing reflection and considering all angles.
		**Application**	There were elements of the framework they would use every time, but not necessarily as a formal process. Specificity of questions was again raised, as was shortening the stakeholder list in the harm–benefit analysis. Coming to a conclusion based only on papers/reports without all the facts was again raised in this comment section. One participant stated, “I prefer the traditional hypothesis, literature search, questionnaire and conclusion approach”.

## Data Availability

Data are contained within the article.

## Supplementary Materials

The following supporting information can be downloaded at: https://www.mdpi.com/article/10.3390/ani13111821/s1, Table S1: Predetermined ethical questions for participants used in each round by discipline. Document S1: Participant questionnaire. Document S2: Round 1 ‘how-to’ framework guide with worked example. Document S3: Round 1 ‘how to access and save the survey’ guide. Document S4: Round 2 ethical framework application document. Document S5: Round 3 ethical framework application document. Document S6: Round 3 worked example. References [85,86,87,88,89,90,91,92,93,94,95,96,97,98] are cited in the supplementary materials.
