# Supplementary material for: Testing and Refining the Ethical Framework for the Use of Horses in Sport
Source: Animals (Basel). 2023 May 31;13(11):1821. doi: 10.3390/ani13111821 (PMC10252045; doi:10.3390/ani13111821)
Supplement: Supplementary file 1 [file animals-13-01821-s001.zip › Document S1 Participant questionnaire.pdf]

Royal Veterinary College
University of London

Questionnaire

On the next page, you will find the questionnaire. Please fill this out AFTER you have completed the framework survey. It is important that you fill out this questionnaire as this is the information we require to modify the framework into a more useable tool. If you have any queries or concerns please email bbrown20@rvc.ac.uk or mcampbell@rvc.ac.uk.

WorldHorseWelfare

SECTION 1: PLEASE PROVIDE SOME INFORMATION ABOUT YOURSELF BY ANSWERING THE FOLLOWING QUESTIONS:

1. How long have you been involved with the equine industry? *(please place an x by your answer)*

- Under 5 years
- 5-10 years
- 10-15 years
- 15+ years

2. What is your main role within equine industry? *(please place an x by your answer):*

- Rider/jockey/driver (competitor)
- Trainer/coach
- Owner
- Groom
- Vet
- Ambassador
- Regulator
- Sponsor
- Competition organiser
- Young rider
- Animal welfare organisation
- Academia/education/research
- Breeder

3. Is this role your main source of income? *(please place an x by your answer)*

- Yes
- No

4. In which discipline do you perform your main role as you have identified it in Q2? *(please place an x by your answer; if your role is not discipline specific, please place an x next to all that apply)*

- Dressage
- Show jumping
- Eventing
- Para dressage
- Para driving
- Flat racing
- Jump racing
- Endurance
- Polo
- Vaulting
- Driving
- Reining

5. How old are you? *(please write your answer below this question)*

……………….. years old

6. Do you identify as *(please place an x by your answer)*

- Male
- Female
- Other
- Prefer not to say

SECTION 2: THE QUESTIONS IN THIS SECTION RELATE TO YOUR TRIAL USE OF THE ETHICAL FRAMEWORK FOR THE USE OF HORSES IN COMPETITIVE SPORT. PLEASE ANSWER EACH QUESTION BY PLACING AN X IN THE BOX UNDERNEATH THE PHRASE THAT YOU MOST AGREE WITH (STRONGLY DISAGREE TO STRONGLY AGREE). THERE IS A COMMENT BOX. PLEASE USE THE COMMENT BOX TO FILL IN ANY COMMENTS WHICH YOU HAVE RELATING TO THE QUESTION / WHICH EXPLAIN WHY YOU CHOSE THE PHRASE WHICH YOU DID.

7. 'I understood how to complete each part of the framework'

Strongly disagree	Mostly disagree	Somewhat disagree	Neither agree nor disagree	Somewhat agree	Mostly agree	Strongly agree

COMMENTS

8. 'I understood all the terms used in the framework'

Strongly disagree	Mostly disagree	Somewhat disagree	Neither agree nor disagree	Somewhat agree	Mostly agree	Strongly agree

COMMENTS

9. ‘The ‘worked example’ helped me understand how to use the framework’

Strongly disagree	Mostly disagree	Somewhat disagree	Neither agree nor disagree	Somewhat agree	Mostly agree	Strongly agree

COMMENTS

10. ‘The stakeholder matrix helped me to apply harm: benefit analysis to the question/issue’

Strongly disagree	Mostly disagree	Somewhat disagree	Neither agree nor disagree	Somewhat agree	Mostly agree	Strongly agree

COMMENTS

11. ‘The framework steps enabled me to come to a conclusion on the specified issue’

Strongly disagree	Mostly disagree	Somewhat disagree	Neither agree nor disagree	Somewhat agree	Mostly agree	Strongly agree

COMMENTS

12. ‘I would use this framework to make decisions in the future’

Strongly disagree	Mostly disagree	Somewhat disagree	Neither agree nor disagree	Somewhat agree	Mostly agree	Strongly agree

COMMENTS

SECTION 3: THESE QUESTIONS RELATE TO YOUR EXPERIENCE OF APPLYING THE FRAMEWORK.

13. Did you identify/ experience any conflicts (clashes of interest) while coming to a decision when using the framework? *(please place an x by your answer)*

- Yes
- No

IF YOU ANSWERED NO, PLEASE GO TO QUESTION 16

14. Was the conflict resolved? *(please place an x by your answer)*

- Yes
- No

IF YOU ANSWERED NO, PLEASE GO TO QUESTION 16

15. How did you resolve the conflict? *(please place an x by all those that apply)*

- By applying the central tenets
- By re visiting the evidence
- Other *(please specify)*

OTHER:

16. Please briefly summarise what you liked about the framework:

COMMENTS

17. Please briefly summarise what you think could be improved about the framework:

COMMENTS

Thank you for your contribution!
