# Supplementary material for: Testing and Refining the Ethical Framework for the Use of Horses in Sport
Source: Animals (Basel). 2023 May 31;13(11):1821. doi: 10.3390/ani13111821 (PMC10252045; doi:10.3390/ani13111821)
Supplement: Supplementary file 1 [file animals-13-01821-s001.zip › Document S2 Round 1 'how to' framework guide with worked example.pdf]

Development of an ethical framework tool for the use of horses in competitive sport - framework 'how to' guide

Thank you for agreeing to help us test our ethical framework for the use of horses in competitive sport. The aim of developing such a framework is to provide stakeholders – whether they be regulators, owners, trainers, riders / drivers, vets, legislators, sponsors, members of the public or others – with a tool which they might apply to the consideration of the ethical questions which inevitably arise in relation to (equestrian) sport. It is hoped that the consensual development of this framework will provide stakeholders with a method of addressing ethical issues which can be consistently applied, so promoting transparent and defensible decision and policy making across disciplines (whilst always allowing for inherent differences between sports).

The testing process which you have kindly agreed to be part of is an important part of this consensual process of development, which will enable us to refine the framework based on your comments about how easy it is to use, through a three-stage process. This should result, at the end of the process, in the production of a practical tool which can be used by a range of stakeholders.

Please note that the framework is NOT designed to tell any stakeholder what conclusion they ought to be reaching on any particular issue – rather, it is designed to provide stakeholders with a logical *method* of reaching some conclusion based on a set of guiding principles. Thus, what you are kindly testing is how easy the framework is to use; we are not testing whether the answer which you reach is 'correct' (there is no 'correct' answer).

Please note also that it is an underlying assumption of this framework that the use of horses in sport *at all* **is** ethically justifiable – providing that certain 'central tenets' are adhered to. These tenets have been derived from consideration of the concepts of 'animal lives worth living' and of 'unnecessary and avoidable harms', and the issue of legality. The central tenets are requirements for the following: (a) minimisation of negative welfare effects and maximisation of positive welfare effects, in order to enable

horses to have ‘lives worth living’ (b) identification of and mitigation against avoidable, unnecessary risk and (c) compliance with governing body regulations and the law. Adhering to these tenets is an integral part of applying the framework. The ethical scenarios and / or ethical question which you will be given to test applying the framework to will not therefore address the overarching question of whether the use of horses in sport *at all* **is** ethically justifiable but will focus on particular ethical dilemmas which can arise within equestrian sport *even when* one accepts that the sport itself is ethically justified.

HOW TO TEST THE ETHICAL FRAMEWORK

You will be sent an ethical scenario/ question and asked to test using the framework on that scenario / question. You test the framework by using the following steps, which are explained here first in text and then in diagrammatic form. For round one of the testing, you will be supplied with the ‘evidence’ which you need to use when applying the framework. In rounds two and three, you will need to find your own evidence using the sources available to you and your existing knowledge (because this is the part of the testing process).

A worked example is provided at the end of this document to help you understand how the framework functions. If you have any questions about how to test the framework, please contact Bluebell Brown at bbrown20@rvc.ac.uk.

WRITTEN STEP BY STEP DESCRIPTION OF HOW TO USE THE FRAMEWORK

Step 1. Define the policy issue

- What is the ethical question / issue which requires a policy formulation / decision?
- Are there any sub-questions which need answering?

Step 2. Identify stakeholders and their interests

Examples of stakeholders who might be relevant include (NB not all stakeholders given as examples here may be relevant to every use of the framework in practice).

- Equine stakeholders
    - Those directly (actively) involved in the sport
    - Those indirectly involved in the sport e.g. young horses not yet in training; retired horses; brood stock; future generations who might be affected by the decision (e.g. through genetic effects)
    - Horses not involved in the sport (if there are relevant 'knock on' consequences)
- Human stakeholders
    - Horse owners
    - Horse breeders
    - Horse riders / jockeys / drivers ('athletes')
    - Those directly employed by or with a business interest in the sport
    - Those indirectly employed by or with an indirect business interest in the sport
    - Veterinary surgeons and other members of the 'veterinary team'
    - Members of the public with an active interest in the sport (e.g. spectators / those engaged in betting)

- Members of the public with no particular interest in the sport but a general interest in animal welfare
- Regulators
- Policy makers
- Law makers
- Animal charities
- Lobbying organisations
- Media

- Other stakeholders
    - ‘The environment’

Step 3. Assess the relevant evidence

- What evidence about the issue under consideration is available?

    - Peer-reviewed journal papers
    - Non-peer reviewed papers, books, and reports
    - Peer reviewed or non-peer reviewed papers which are not about the issue under consideration but are about a related issue (for example in other species, or other sports)
    - Expert opinion
    - Stakeholder opinions (e.g. from publications; conference proceedings, websites etc)

Consideration should be given to the quality of evidence

- What evidence about the issue under consideration is lacking / how could this be obtained?

Step 4. Identify relevant legislation / regulation

- International legislation
- National legislation
- Sport specific regulations (which may include international or national variation)

Step 5. With reference to the interests of each stakeholder and considering also the severity and duration of impact on stakeholders, apply **a harm: benefit analysis to the question / issue.**

Use of a 'stakeholder matrix' such as this one may help to focus this consideration:

**Stakeholder**	**Harms associated with the action / decision**	**Benefits associated with the action / decision**
1		
2		
3		
4		
etc		

- Reach preliminary conclusion / decision based on the harm: benefit analysis

Step 6. Test preliminary conclusion / decision against the central tenets

The central tenets of the framework are:

- Minimisation of negative welfare effects and maximisation of positive welfare effects for horses.
- Identification of and mitigation against avoidable, unnecessary risk to horses.
- Compliance with governing body regulations and the law.

If any of the central tenets are compromised by the preliminary conclusion / decision reached through the harm: benefit analysis, reassess both the analysis and the conclusion.

NB: There may be occasions on which the initial conclusion is not compliant with current regulation / legislation and when - having reassessed both the analysis and the conclusion – the users of the framework still believe that their conclusion is correct and that current regulation / legislation needs reviewing. If this occurs it should be explicitly stated and recorded.

Note: this testing against central tenets will assist in 'weighing' different stakeholder interests if a particular conclusion / decision would, in a harm: benefit analysis, be to the overall benefit of one stakeholder and the detriment of another. For example, a particular preliminary conclusion / decision from the harm: benefit analysis might provide a substantial economic benefit to many humans but involve the acceptance of an identifiable risk to equine welfare which could be mitigated against. In that case, implementing the preliminary conclusion / decision would contravene one of the central tenets of the framework ('Identification of and mitigation against avoidable, unnecessary risk to horses'). This would indicate that the weighting of interests in the conclusion of the harm: benefit should be shifted in favour of equine (not human) interests, and the conclusion adjusted accordingly. Thus, testing initial conclusions from the harm: benefit analysis against the central tenets is a balancing and rebalancing process. The framework deliberately does not say anything about the relative weighing of different (sometimes conflicting) human interests – that must be left to the users of the framework, with appropriate acknowledgment of conflicts where they occur (see below).

Step 7. Identify any conflicts in the conclusion / decision.

Conflicts may occur between stakeholder interests, or in the acceptance amongst those using the matrix of the conclusion / decision which has emerged from its employment.

- Can any conflicts be resolved by further reference to the central tenets of the framework (see 'testing against central tenets' above)?

- Can any conflicts be resolved by reference to evidence? Sometimes, apparent conflicts of interest are in fact disagreements over facts and can be resolved by elucidation of those facts or by gathering further evidence (for example, about the extent or nature of a harm).

- It is to be expected that conflicts will occur. Where this happens and they cannot be resolved they should be noted, along with a brief explanation of the reason why they cannot be resolved (e.g. insufficient evidence to reach a definitive conclusion; disagreement about weighing interests etc).

Step 8. Agree final conclusion / decision / outcome

- Record any dissenting opinions
- Note any further work which needs to be done (e.g. to gather further evidence)
- Agree an action plan to be implemented as a consequence of the conclusion / decision

Step 9. Agree a plan for future review of the decision

For example, if a lack of evidence has been identified as a factor limiting the validity of the decision, make a plan for commissioning appropriate research / tracking the publication of relevant evidence and reviewing the decision when the evidence does become available.

Please see the next page for the diagrammatic explanation of how to use the framework.

Diagrammatic step by step explanation of how to use the ethical framework

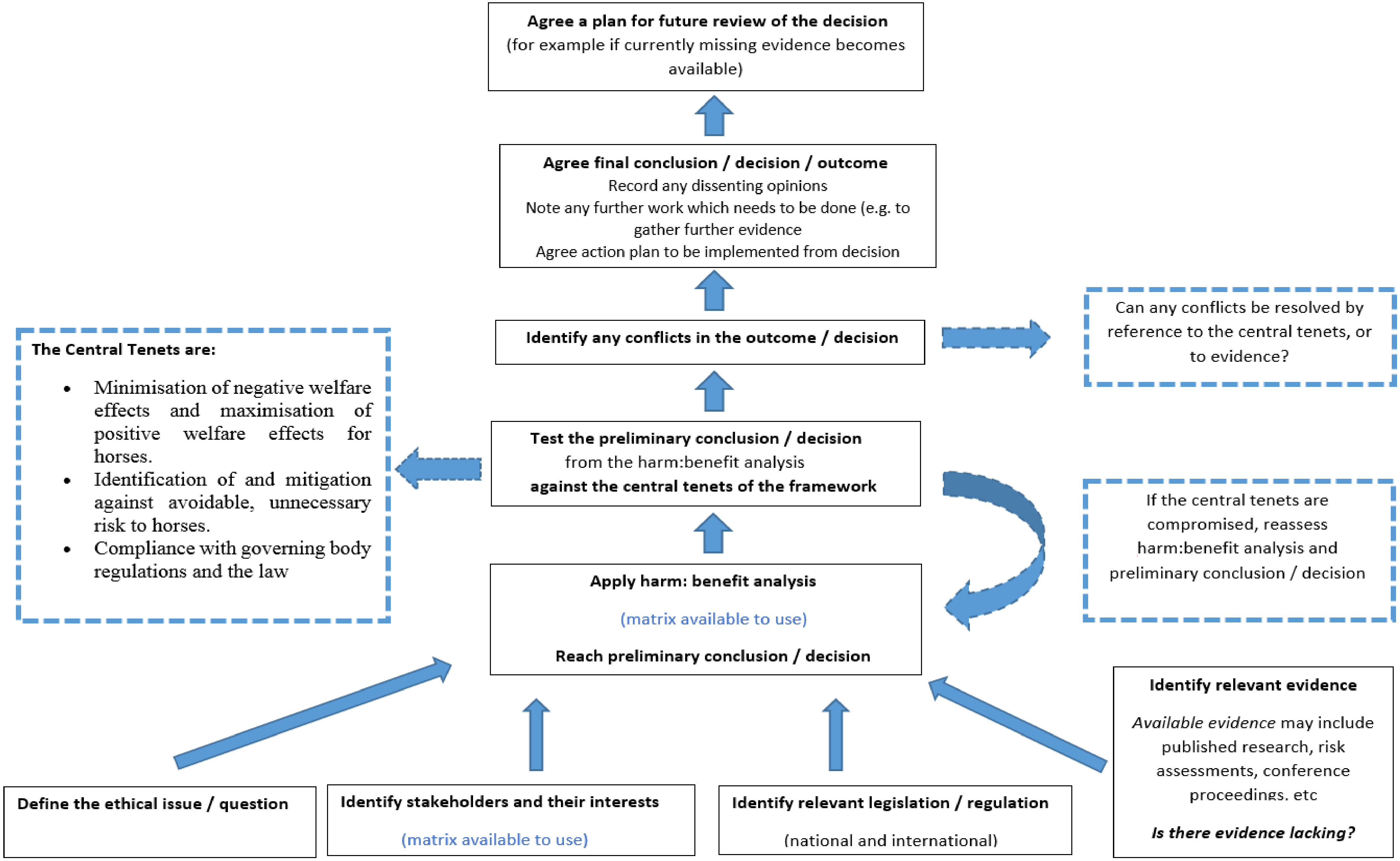

A WORKED EXAMPLE OF USING THE ETHICAL FRAMEWORK FOR THE USE OF HORSES IN COMPETITIVE SPORT.

Recently, there has been interest in the lay equine press in the question of whether all horses being kept in a stable require daily turnout. Whilst such equine management is legislated for in some countries, in others daily turnout is a recommendation rather than a requirement. This worked example uses that issue as an example question with which to demonstrate the use of the framework. The purpose of providing this worked example is not to suggest a 'correct' answer to this particular question, but simply to demonstrate how the framework itself could be used to approach *any* ethical question.

Step 1. Define the policy issue

- What is the ethical question / the issue which requires a policy formulation / decision?

The more general question of whether all horses kept in stables should be turned out for free exercise is narrowed to apply to competition horses only, since this is an ethical framework for the use of horses in competitive sport. Thus, the ethical question is determined to be: '**Should it be a legal requirement that all horses primarily used for competitive purposes which are kept in stables be turned out for free exercise on a daily basis?'**

NB this does not apply to horses which are being kept stabled under veterinary advice (e.g. due to injury).

NB 'competition' is defined as any horses involved in any equestrian sport or being raised for a future in or retired from such involvement.

NB 'turnout' in this context is being considered purely in relation to the ability to exercise and express normal behaviours. For ease of analysis, it is assumed that adequate nutrition will be provided whether or not the horse is turned out.

- Are there any sub-questions which need answering?

Could also consider whether horses should always be turned out with company (either equine or another animal) or whether it is acceptable to turn them out alone.

Could also consider whether horses of differing ages and sexes have different needs.

(For reasons of space, these supplementary questions will not be considered in detail in the worked example).

Step 2. Identify stakeholders and their interests

Examples of stakeholders who might be relevant include:

- Equine stakeholders

All competition horses at all stages of life who are being kept stabled.

- Human stakeholders
    - Horse owners
    - Horse breeders
    - Those directly employed by or with a business interest in equestrian sport for which horses are kept for competition e.g. grooms, riders, trainers, regulators for a given sport.
    - Those indirectly employed by or with an indirect business interest in equestrian sport for which horses are kept stabled or kept outside of stables e.g. manufacturers of stables; bedding suppliers; fencing suppliers; muck heap removal services.
    - Veterinary surgeons and other members of the 'veterinary team'
    - Members of the public with an active interest in the sport (e.g. spectators / those engaged in betting).
    - Members of the public with no particular interest in the sport but a general interest in animal welfare.
    - Regulators
    - Policy makers
    - Law makers
    - Animal charities
    - Lobbying organisations
    - Media

- Other stakeholders
    - ‘The environment’ (impact via poaching of fields, muck removal, water supply etc).

Step 3. Assess relevant evidence

- What evidence about the issue under consideration is available?
    - Peer-reviewed journal papers

There is extensive literature about the relationship between being stabled and the development of abnormal (stereotypic) behaviours in horses, e.g. [2-8]. Providing exercise, including in the form of turnout, generally reduces behaviours which are undesirable during standard handling / training situations [9].

Much less has been published about the relationship between keeping horse either stabled or turned out and the incidence of and injury and disease [7].

There is published literature to support the idea that exercise in young horses offers a protective effect against some forms of orthopaedic disease and fatality due to injury [10-14]. However, these studies assessed the effects of human-controlled exercise rather than of free exercise in a turn out area. The protective effect of exercise during turnout is therefore likely but not proven.

- Non-peer reviewed papers, books, and reports

A number of such articles exist which in recent years have tended towards recommending daily turnout whilst recognising the risks of injury which can be associated with it, e.g.

- Peer reviewed or non-peer reviewed papers which are not about the issue under consideration but are about a related issue (for example in other species, or other sports)

See notes above re peer reviewed articles on the protective effect of controlled exercise

- Expert opinion

The UK's Code of Practice for the Welfare of Horses, Donkeys and Hybrids recommends that horses should be turned out.

- Stakeholder opinions (e.g. from publications; conference proceedings, websites etc)

Several websites of equine welfare charities / organisations recommend daily turnout whilst recognising the risks of injury which can be associated with it e.g. https://www.worldhorsewelfare.org/advice/management/stabling
https://www.rspca.org.uk/adviceandwelfare/pets/horses/behaviour
https://www.bhs.org.uk/advice-and-information/horse-care/winter-care

- What evidence about the issue under consideration is lacking / how could this be obtained?

Information about incidence and causes of disease and injury in horses which are kept stabled except when undertaking controlled exercise compared to those which have a daily opportunity to exercise freely is lacking. Research is necessary to provide this information.

Step 4. Identify relevant legislation / regulation

- International legislation
- National legislation
- Sport specific regulations (which may include international or national variation)

International legislation:
OIE standards on *animal welfare do not address competition horses (though they do address 'working equids').*
Animals used in competitions are excluded from EU Treaties.
In Switzerland, the Animal Welfare Ordinance of 2008 https://www.globalanimallaw.org/downloads/database/national/switzerland/TSchV-2008-EN-455.1-2011.pdf dictates that working horses (those ridden or worked regularly) must be allowed free time in open outdoor areas at least two days a week

for at least two hours each time. Unworked horses (e.g./ retired horses or broodmares) must have at least two hours of outdoor free time every day. Young horses (up to two-and-a-half years old) must be kept in groups.
Danish law requires paddocks to be available at all premises which keep horses.

National legislation (for the purposes on this worked example we concentrate on UK legislation):
The Animal Welfare Act (2006) (England and Wales), the Animal Health and Welfare (Scotland) Act 2006 and the Welfare of Animals (Northern Ireland) Act 2011 require owners and keepers to ensure that horses' needs to express normal behaviours are met.
The Code of Practice for the Welfare of Horses, Donkeys and Hybrids suggests that 'All stabled horses, apart from those on box rest for veterinary reasons, will benefit from daily turnout'

Sport specific regulations (which may include international or national variation):
None found

Step 5. With reference to the interests of each stakeholder and considering also the severity and duration of impact on stakeholders, **apply a harm: benefit analysis to the question / issue**

Gather information about how many competition horses are likely to be affected by this decision.

Horses who are kept stabled all of the time except when being exercised under the control of a human are likely to suffer chronic, low - moderate level harms (e.g. boredom; stiffness, chronic respiratory disease) and may sometimes suffer more acute, higher intensity harms (e.g. colic). Horses who are turned out daily (especially if weather conditions are inclement) may suffer chronic, low level harms (e.g. 'mud fever') or acute, more intense harms e.g. orthopaedic injury. See matrix overleaf. Basic needs of horses include:

- Freedom from discomfort
- Freedom from pain, injury and disease
- Freedom to express normal behaviour

Use of a 'stakeholder matrix' such as this one may help to focus this consideration:

NB In this example, for reasons of space, the matrix has not been comprehensively completed - example key stakeholders only have been included, for the purposes of illustration.

**Stakeholder**	**Harms associated with the action / decision**	**Benefits associated with the action / decision**
1 Horses	Being kept stabled restricts the freedom to express normal behaviours and to move normally and may result in stereotypic behaviours/ 'stable vices'.<br><br>Being kept stabled is associated with an increased incidence of some diseases e.g. respiratory disease; colic.<br><br>Daily turn out may cause injuries e.g. through entanglement in fencing, uncontrolled exercise or interaction with other horses.<br><br>Weather conditions e.g. excessive heat or rain may result in discomfort during turnout.	Daily turnout facilitates expression of normal behaviours and freedom of movement.<br><br>Daily turnout *may* help to reduce the risk of orthopaedic injury during training and competition by keeping animals supple and building muscle strength and co-ordination

2 Owners	Variable financial impact of keeping horses stabled versus stabled plus daily turnout.<br><br>Being kept stabled may result in increased veterinary costs for issues such as respiratory disease.<br><br>Daily turnout could increase veterinary costs IF more injuries resulted.<br><br>IF more injuries resulted from turnout then competitive career and value of horse could be negatively affected.<br><br>In some parts of the world it is difficult to provide suitable turn out during very wet winters. If weather conditions are inclement horse may be more prone to conditions such as 'mud fever', resulting in increased veterinary costs<br><br>Owners may feel that regulation requiring daily	Horses may be less stressed as a result of daily turnout, with positive effects e.g. reduction in gastric ulceration (which reduces veterinary costs and improves competitive performance).<br><br>IF daily turnout is associated with a protective effect against orthopaedic injury / disease then competitive career and value of horse could be positively affected.<br><br>Owner may 'feel good' about their horse having the freedom to exhibit more natural behaviours

	turnout of horses is 'interference' in their autonomous right to look after their property as they see fit.	
3 Grooms	Horses which are turned out daily are likely to need more grooming; increased work associated with wet rugs and leading horses to and from fields, supplying feed in field etc.	Less time stabled may reduce workload of mucking out.<br><br>Groom may 'feel good' about their horse having the freedom to exhibit more natural behaviours.<br><br>Horses may be easier to handle if they have time to exercise freely.
4 Rider	Having horses turned out may be less convenient and more time consuming than having them readily accessible in a stable.	Horses who have been relaxing and moving around during turnout may be more easily trainable and require less warm up time than those who are always kept stabled.
5 Public with an interest in animal welfare		Interested in animals' freedoms to express normal behaviours being protected. May feel that voluntary adoption of good practice is insufficient, and that

		legislation is necessary to safeguard animal welfare.
6. Policy makers (government)	Government may have an ideological objection to 'interfering' in animal owners' autonomous decision-making processes.<br><br>Legislation may not necessarily be the most effective means of affecting desirable changes in equine management processes.<br><br>Legislation is only effective if enforced, which requires financial commitment.	Animal welfare is generally a vote winning (or losing) issue in some countries. Governments may thus have a pragmatic interest in visibly driving national animal welfare laws which reflect public attitudes towards animal welfare.<br><br>Governments may have an ideological commitment to safeguarding animal welfare.
Etc		

Summary of harm: benefit analysis

Daily turnout for stabled competition horses is associated with clear benefits to psychological animal welfare and behaviour. Daily turnout may also be associated with some risks to animal welfare through turn-out associated injury and disease. However, being kept stabled is also associated with (different) risks to health and therefore welfare, and additionally has a negative psychological effect on welfare.

Allowing for uncertainty arising from the weighing of these risks and benefits to equine health associated with being kept stabled versus being turned out, the advantages to all human stakeholders or keeping horses stabled are primarily those of convenience,

and the disadvantages of daily turn out are primarily those of inconvenience (which may be associated with increased staff costs).

There are precedents showing that any concerns on the part of animal owners about contravention of their autonomy and right to make decisions about how they keep their property (animals) may be overridden by animal welfare interest – animal welfare legislation already very clearly establishes limitations to ways in which owners may treat animals, and these limitations are supported by welfare codes of practice.

'The public' has an interest in animal welfare being safeguarded and would therefore support systems which enable the expression of normal behaviours.

Policy makers have an interest in regulations pertaining to horses being consistent with national and international animal welfare laws and reflecting public attitudes towards animal welfare and are therefore likely to support systems which enable the expression of normal behaviours.

- Reach preliminary conclusion / decision based on the harm: benefit analysis

There is a lack of evidence about rates of injury and disease in horses kept stabled versus those kept stabled with daily turnout, which needs to be addressed.

Daily turnout may be associated, through impact on injury and disease, with some negative welfare effects for individual horses.

There is clear evidence that keeping horses stabled all the time has a detrimental effect on welfare by limiting opportunities for normal behaviours. Daily turnout is likely to be associated with positive psychological welfare effects for the vast majority of horses.

Based on current evidence, it therefore seems likely that daily turnout of competition horses would improve equine welfare overall and be consistent with the interests of members of the public who are interested in animal welfare, and of government.

Harms to owners / riders / grooms do exist but are largely those of inconvenience and therefore outweighed by the benefits to horses, the public and governments.

Improvements in animal welfare may be affected either through legislation, regulation of voluntary adoption of recommendations. It is suggested that laws which positively state what a person must do are most effective in terms of improving animal welfare. However, any legislation is only effective if enforced.

Preliminary conclusion: it should be a legal requirement that stabled competition horses should be turned out daily. However, the effectiveness of adopting such a policy is dependent upon having the resource to enforce any new legislation.

Step 6. Test against the central tenets

The central tenets of the framework are:

- Minimisation of negative welfare effects and maximisation of positive welfare effects for horses.

As described above, daily turnout may be associated, through impact on injury and disease, with some negative welfare effects for individual horses. However, daily turnout is likely to be associated with positive psychological welfare effects for the vast majority of horses. The preliminary decision is therefore consistent with this tenet.

- Identification of and mitigation against avoidable, unnecessary risk to horses.

Many of the risks associated with daily turnout e.g. of injury due to fencing and interaction with other horses and disease due to muddy conditions can be mitigated against by management systems. Such mitigation should recognise, for example, the fact that safety may be increased by turning some horses out by themselves (e.g. stallions, or if one horse persistently kicks other horses); and that horses who are not used to being turned out may find it initially stressful and should be introduced to turn out for short, increasing periods.

The preliminary decision is therefore consistent with this tenet and considered application of this tenet in fact shifts the harm: benefit analysis further in the direction of concluding that daily turnout should be a legal requirement.

- Compliance with governing body regulations and the law

The preliminary decision is consistent with existing legislation e.g. The Animal Welfare Act (2006) (England and Wales), the Animal Health and Welfare (Scotland) Act 2006 and the Welfare of Animals (Northern Ireland) Act 2011. Specific regulation / secondary legislation may be necessary to implement the conclusion of this analysis.

- If any of the central tenets are compromised by the preliminary conclusion / decision reached through the harm: benefit analysis, reassess both the analysis and the conclusion.

Not necessary.

Step 7. Identify any conflicts in the conclusion / decision.

Conflicts may occur between stakeholder interests, or in the acceptance amongst those using the matrix of the conclusion / decision which has emerged from its employment.

- Can any conflicts be resolved by further reference to the central tenets of the framework? (see 'testing against central tenets' above.)

Conflicts between the needs of horses and the convenience-based interests of owners / grooms / riders may be resolved through application of the tenets, as described above.

There is a conflict between owners' autonomous rights to make their own decisions about how their property is managed, and the requirement to meet animals' basic needs. Childress *et al* [1] suggest that where such conflicts exist the right to autonomy has to be qualified – giving rise to a principle of 'least infringement'.

Conflicts inherent within the interests of horses e.g. risk of disease higher if kept in, risk of injury higher if turned out, may be reduced through application of the tenets, as described above.

- Can conflicts be resolved by reference to evidence? Sometimes, apparent conflicts of interest are in fact disagreements over facts and can be resolved by elucidation of those facts or by gathering further evidence (for example, about the extent or nature of a harm).

Potential conflicts e.g. increased veterinary costs for owners associated with turn out versus improved psychological welfare for horses associated with turnout may be resolved in future by acquisition of further evidence about incidence and causes of disease and injury in horses which are kept stabled except when undertaking controlled exercise compared to those which have a daily opportunity to exercise freely
.

Step 8. Agree final conclusion / decision / outcome

- Record any dissenting opinions
- Note any further work which needs to be done (e.g. to gather further evidence)

Further work needed to gather evidence about the risks to equine health associated with being turned out and stabled. Note that these risks may differ for individual horses, depending on temperament and previous experience.

Further work needed to provide evidence about the optimal minimum time of daily turnout for horses – it is not clear where such specifications as exist in international legislation originate.

- Agree an action plan to be implemented as a consequence of the conclusion / decision

Decision that daily turnout for competition horses should become a legal requirement agreed. HOWEVER, further work is now needed to elucidate the most effective means of affecting desirable changes in equine management processes (e.g. secondary

legislation) and to determine what minimum daily time period of turn out (if any) should be specified.

Step 9. Agree a plan for future review of the decision

For example, if a lack of evidence has been identified as a factor limiting the validity of the decision, make a plan for commissioning appropriate research / tracking the publication of relevant evidence and reviewing the decision when the evidence does become available.

Publication of evidence about the risks to equine health associated with being turned out and stabled to be tracked. Decision to be reviewed in light of future publications.

Before the policy recommendation can be implemented, further work is needed to elucidate the most effective means of enacting this policy recommendation (e.g. through secondary legislation).

Before the policy recommendation can be implemented, the basis of minimum daily time period of turn out (if any) which would be specified in legislation needs to be explained.

Reference List

1. Childress, J.F., et al., *Public health ethics: mapping the terrain.* J Law Med Ethics, 2002. **30**(2): p. 170-178

2. Cooper, J.J. and G.J. Mason, *The identification of abnormal behaviour and behavioural problems in stabled horses and their relationship to horse welfare: a comparative review.* Equine Vet J Suppl, 1998(27): p. 5-9.

3. Henderson, A.J.Z., *Don't Fence Me In: Managing Psychological Well Being for Elite Performance Horses.* Journal of Applied Animal Welfare Science, 2007. **10**(4): p. 309-329.

4. Burger, D., et al., *Applied research on equine behaviour.* Revue Suisse d'Agriculture, 2008. **40**(3): p. 109-115.

5. Benhajali, H., et al., *Foraging opportunity: a crucial criterion for horse welfare?* Animal, 2009. **3**(9): p. 1308-1312.

6. Minero, M. and E. Canali, *Welfare issues of horses: an overview and practical recommendations.* Italian Journal of Animal Science, 2010. **8**(1s): p. 219.

7. Keeling, L., E. Hartmann, and E. Søndergaard, *Keeping horses in groups: A review.* Applied Animal Behaviour Science, 2012. **136**(Issue 2-4): p. 77-87.

8. Tadich, T., et al., *Husbandry practices associated with the presentation of abnormal behaviours in Chilean Creole horses.* Archivos de Medicina Veterinaria, 2012. **44**(3): p. 279-284.

9. Freire, R., P. Buckley, and J.J. Cooper, *Effects of different forms of exercise on post inhibitory rebound and unwanted behaviour in stabled horses.* Equine Vet J, 2009. **41**(5): p. 487-92.

10. Smith, R.K., et al., *Should equine athletes commence training during skeletal development? changes in tendon matrix associated with development, ageing, function and exercise.* Equine Veterinary Journal, 1999. **31**(S30): p. 201-209.

11. Firth, E.C. and C.W. Rogers, *Musculoskeletal responses of 2-year-old Thoroughbred horses to early training. Conclusions.* N Z Vet J, 2005. **53**(6): p. 377-83.

12. Firth, E.C., *The response of bone, articular cartilage and tendon to exercise in the horse.* J Anat, 2006. **208**(4): p. 513-26.

13. Verheyen, K.L., *Reducing injuries in racehorses: mission impossible?* Equine Vet J, 2013. **45**(1): p. 6-7.

14. Rogers, C.W., et al., *Evaluation of a new strategy to modulate skeletal development in Thoroughbred performance horses by imposing track-based exercise during growth.* Equine Vet J, 2008. **40**(2): p. 111-8.
