# Supplementary material for: Testing and Refining the Ethical Framework for the Use of Horses in Sport
Source: Animals (Basel). 2023 May 31;13(11):1821. doi: 10.3390/ani13111821 (PMC10252045; doi:10.3390/ani13111821)
Supplement: Supplementary file 1 [file animals-13-01821-s001.zip › Document S3 Round 1 'how to access and save the survey' guide.pdf]

How to access and save the 'Development of an ethical framework tool for the use of horses in competitive sport' survey

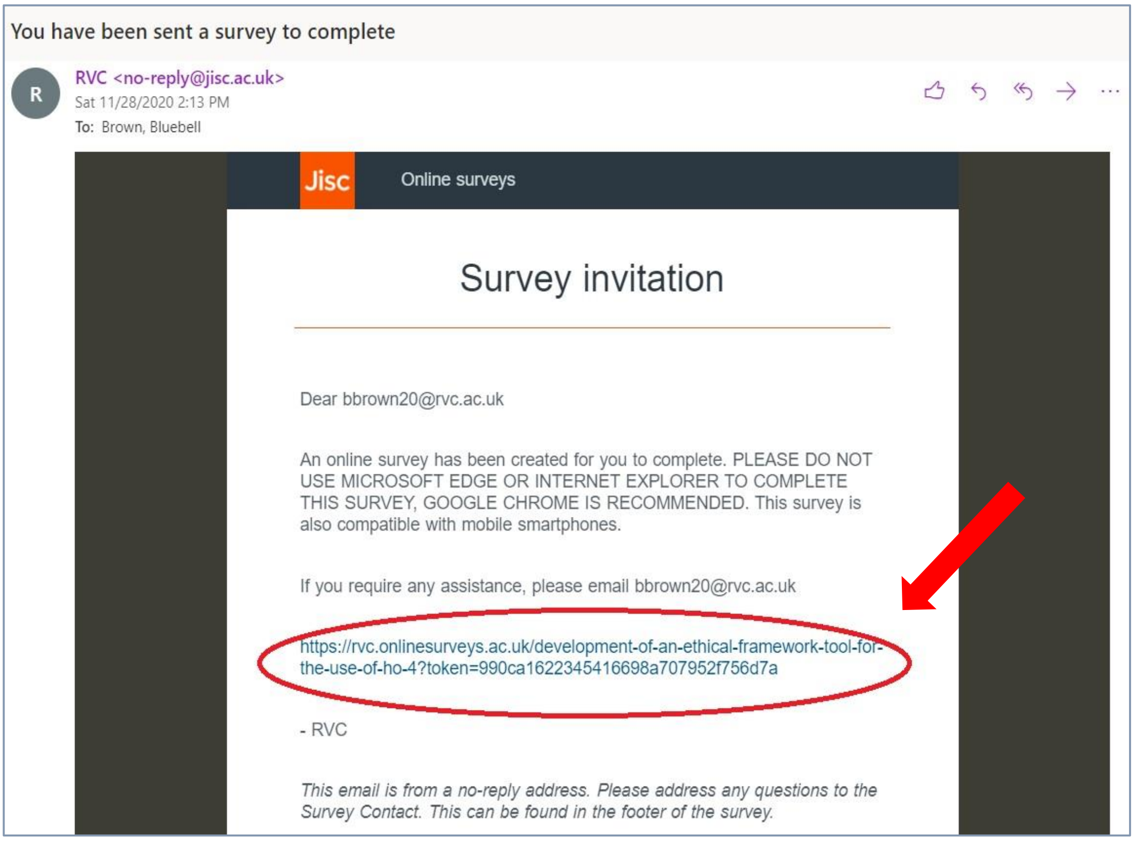

You will receive an email invite. Click on your personal survey link that is highlighted in red.

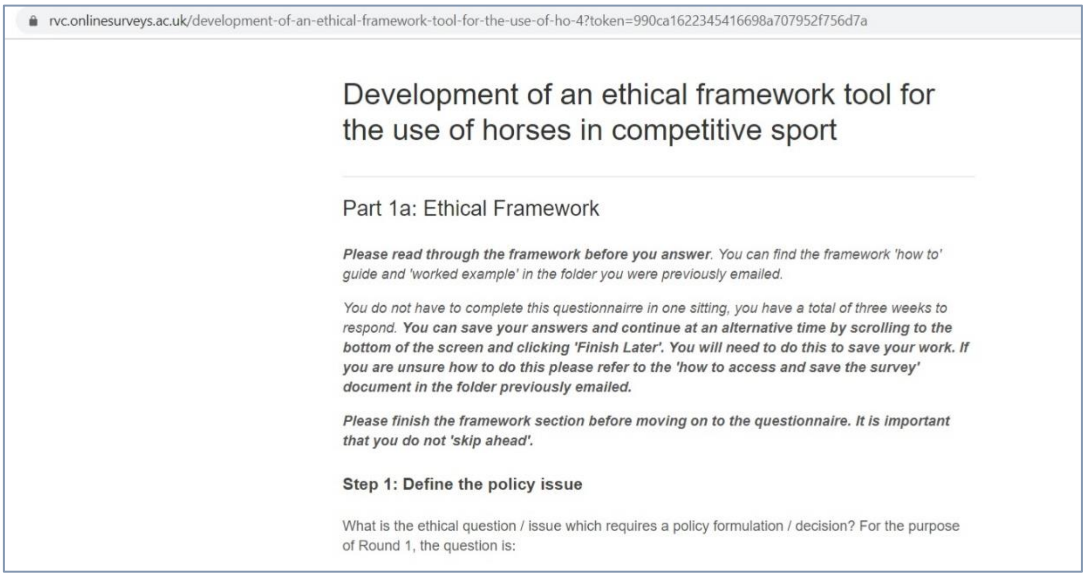

You will be taken to the survey page and can start completing the questions.

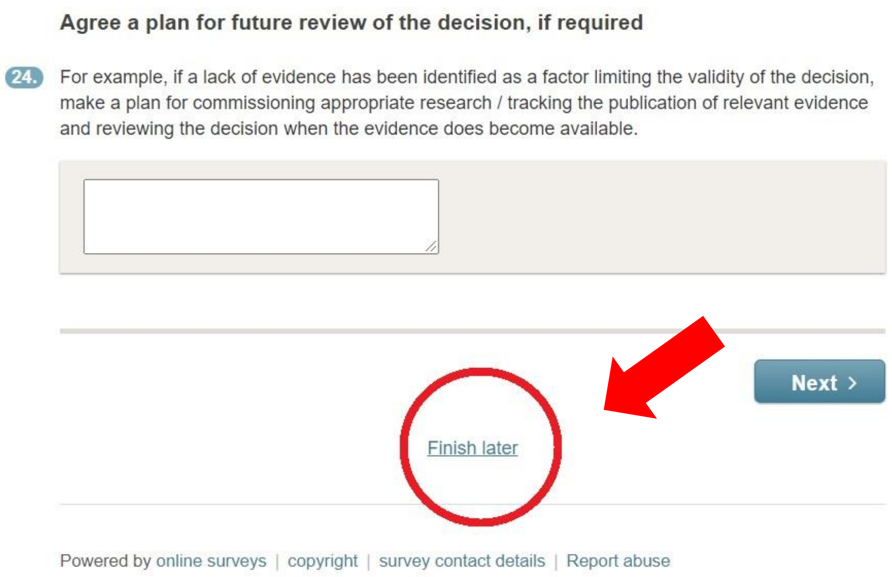

If you would like to continue with the survey at an alternative time, scroll to the bottom of the page and click ‘Finish later’ to save your work.

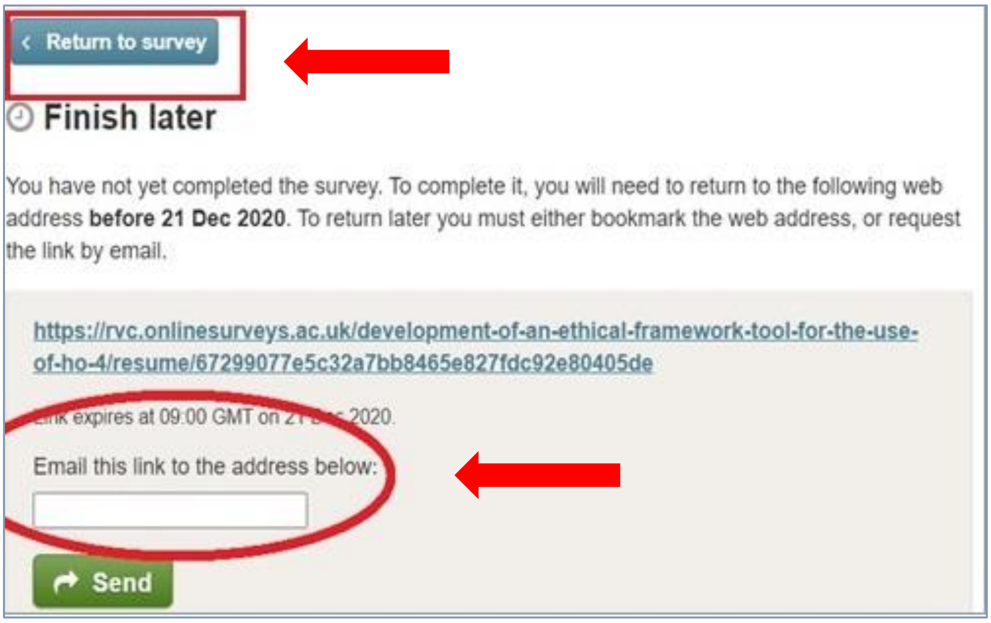

When you click finish later, this screen will appear. Enter your email address in the highlighted area and press send. Alternatively, you can ‘bookmark’ the page if you prefer and return to the survey from wherever you saved your bookmark. This screen will reappear – click ‘return to survey’ in the left -hand corner.

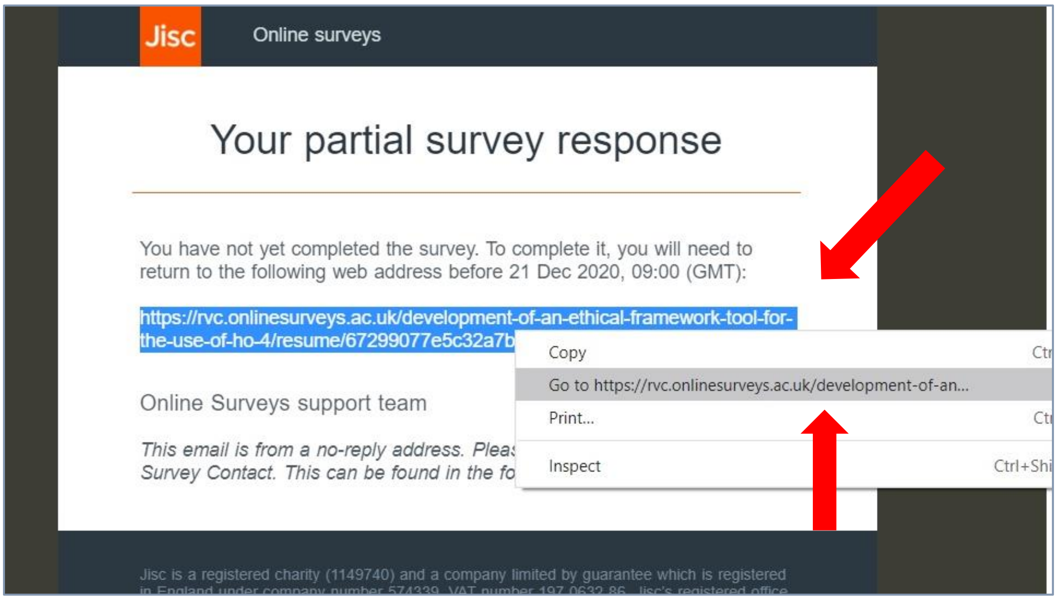

If you chose the email option, you will receive an email like this. Highlight the link with your mouse, right click on ‘go to https://...’. This will reopen the survey.
