# Supplementary material for: Testing and Refining the Ethical Framework for the Use of Horses in Sport
Source: Animals (Basel). 2023 May 31;13(11):1821. doi: 10.3390/ani13111821 (PMC10252045; doi:10.3390/ani13111821)
Supplement: Supplementary file 1 [file animals-13-01821-s001.zip › Document S4 Round 2 ethical framework application document.pdf]

Development of an ethical framework tool for the use of horses in competitive sport

'Round 2' framework survey

Table of Contents

Putting the framework in context - video	i
Document guide	i
Participant framework survey	1
- Question & background	2
- Sport rules/laws	3
- Evidence	5
- Stakeholder harm: benefit analysis	8
- Your preliminary decision	10
- The Central Tenets	11
- Acknowledging and resolving conflict	12
- Final decision	13
Questionnaire	15
Appendix A<br>- Worked Example	23

RVC Royal Veterinary College University of London

World Horse Welfare

Putting the framework in context – introductory video

A short introductory video for this round of testing is available for you to view if you wish to do so: please click on or copy and paste this link into your internet browser https://youtu.be/rl_tHSd5Z_4

Instructions on completing the survey as a Word document

For Round 2, the framework survey and questionnaire have been provided as a Word document. This is to help make access and completion easier for participants. You have three weeks to complete the framework survey and questionnaire. Please email your completed document by the 12th of March 2021 to bbrown20@rvc.ac.uk. Please also:

- REMEMBER to SAVE YOUR WORK!
- Do not alter the document, apart from inserting your responses in the text boxes provided. To do this, place your mouse in the box, click and start typing.

THANK YOU FOR YOUR PARTICIPATION

Participant framework survey

On the next page, the framework survey starts. This begins with a discipline specific question for you to consider. Follow the steps and guidance provided to complete the framework. If you have any queries or concerns please email bbrown20@rvc.ac.uk or mcampbell@rvc.ac.uk.

1 The Question is:

Should Young Horse Classes be allowed?

Background

Young Horse Classes are popular within the industry. Some concerns have been expressed about the impact of preparing horses for such classes on medium-long term health and welfare.

2 Sport rules / laws:

FEI:
https://inside.fei.org/content/general-regs-statutes

POLO:
https://hpa-polo.co.uk/

RACING:
http://rules.britishhorseracing.com/#!/book/34

To determine whether something should be 'allowed' or 'not allowed' during competition and competition training, we first need to know what current rules, regulations, and legislation there are on this issue. Rules for each discipline are created by the regulatory body, both international and national. For example, FEI (international) covers all disciplines within this study, except racing and polo. There may be relevant rules in the general / veterinary / discipline specific regulations and there is also the FEI Code of Conduct. You can look up the rules for each discipline by clicking the relevant links on the left hand side of this page or by copying and pasting those links into your internet browser. Note that participants in Round 2 testing are being asked different questions from each other and therefore not all of the links shown will be relevant to your discipline / the particular question which you have been asked. Laws can refer to legislation like The Animal Welfare Act, 2006. You can search for legislation via Google or on a government website like gov.uk. Are there any sports rules / laws which are relevant to the question which you are answering? If so, make a brief note in the sport rules / laws box on the next page. It will be helpful to write down who made the regulation (e.g. BHA) and what it is. If you would like to see a completed example of this framework for guidance, go to Appendix A on page 23.

SPORT RULES/LAWS (you are not expected to fill this entire box)

3 Evidence:

In order to make an ethical decision, we need to consider what evidence is available which would support a decision one way or the other. There are several types of evidence, which have varying degrees of quality. The information boxes below will help you to identify different types of evidence and how to find them. You may also already know some evidence about the question which you are considering. In the box on the next page, write down any evidence which you already know or have been able to find about the question, including who the author (organisation or person) was and a brief note on what the evidence said.

RESEARCH ARTICLE. The 'evidence' within a research article is based on the results of some form of scientific research, which is usually assessed by other scientists before it is published. Where possible, you should aim to try and include this as one of your main sources of evidence, as this is considered 'objective' – the researchers do not have a vested interest towards the outcome of the research. To search for a research article online, you can use 'Google Scholar'. If you don't already have this browser, type 'Google Scholar' into Google, click on the result and use this browser to search for information, like you would do for normal Googling. Click on one of the relevant results, this should usually take you to a 'summary' or 'abstract', a short paragraph about the research and its results. If you find relevant information, you can read these summaries and note them down in the 'evidence' box on the next page.

BOOKS, REPORTS, MAGAZINE ARTICLES. You may have read some information in a book or magazine that relates to the question or you can search for this information – in a library, Google books or Google. For magazine articles, TheHorse.com, the FEI website and Horse & Hound are good places to start. These articles are generally based on research findings or are written by an 'expert' in the area, e.g. a vet, but are usually easier to understand than research articles. It might be difficult to find something directly relevant but there may be more information on this area in other species or sports.

STAKEHOLDER EXPERT OPINION/ PERSONAL EXPERIENCE. You may have attended a conference or a seminar about the issue. You can include this within your evidence box. You may also have some personal experience which you would like to include.

EVIDENCE (there is an additional box on the next page if you require extra space)

EVIDENCE (you do not have to fill in this box, it is here if you require extra space.)

4 Stakeholder harm: benefit analysis

In order to come to a decision on whether something is 'right or wrong', a common approach is based on 'utilitarianism', which looks at the consequences of an action. This is where an action or decision is considered ethically 'right' if it achieves the greatest good (or benefit) for the greatest number. Any decision will affect many people through knock on consequences, and so it is important to weigh up the 'harm' and 'benefit' for each 'stakeholder'. A 'stakeholder' is anyone affected by the decision. Below are examples of 'equine' (red) 'human' (blue) and 'other' (purple) stakeholders, that may be impacted by the question under consideration. Consider which stakeholders are likely to be impacted and in the table overleaf (the stakeholder matrix) note down the stakeholder, and the potential 'harm' and 'benefit' to that stakeholder, if the answer to the question which you are considering were 'yes'. Not all stakeholders are relevant in every situation and there may be stakeholders that you identify as impacted that are not listed here. As the goal of this framework it to come to an ethical decision in relation to the *use of horses* in competitive sport, the stakeholder matrix ***MUST include at least one equine stakeholder.***

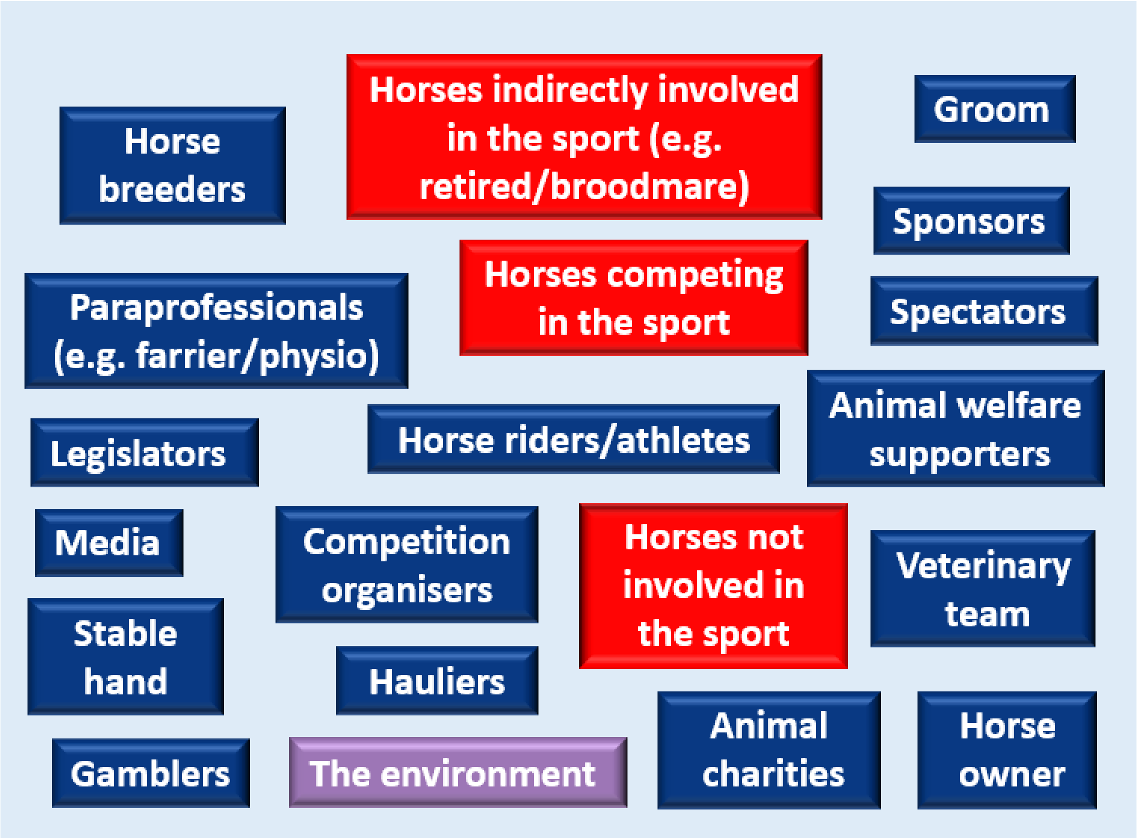

Stakeholder	Potential harm to stakeholder	Potential benefit to stakeholder

5 Your preliminary decision:

In the box below, note down your preliminary answer to the question which you are considering, based on the harm: benefit analysis of stakeholders' interests.

YOUR PRELIMINARY DECISION

6 The Central Tenets:

Your preliminary decision should be tested against the **'central tenets' of the framework** which are listed in the box to the right. This assists 'weighting' different stakeholder interests if the harm: benefit analysis suggests that a particular decision is of benefit to one stakeholder and to the detriment of another. In other words, applying the central tenets to your preliminary decision helps you decide whose interests should be given precedence. For example, suppose that you have gathered the evidence and applied the harm: benefit analysis and made an initial decision in favour of an action which is economically beneficial to humans but also carries an identifiable risk of an avoidable harm to horses. When you check your initial decision against the central tenets, you will see that one of the tenets states that there must be 'identification and mitigation against avoidable, unnecessary risk to horses'. Your initial decision to allow human economic benefits to override equine harms is in contradiction to this tenet and therefore your initial conclusion / decision needs to be reconsidered.

The Central Tenets

Minimisation of negative welfare effects and maximisation of positive welfare effects for horses.

Identification of and mitigation against avoidable, unnecessary risk to horses.

Compliance with governing body regulations and the law.

7 Acknowledging and resolving conflicts:

At the moment, you are testing this framework individually but in Round 3 of testing and thereafter it will normally be used by groups of people working together to consider an ethical issue. Conflicts may occur between the interests of those who are together using the framework to consider a question. Such conflicts may make it difficult for all involved to subscribe to the decision which has been reached by applying this framework. Consider whether, even when you are testing the framework individually, you can identify such conflicts. For example, if you play more than one role in your discipline (perhaps you are both a rider and a breeder or owner) would the conclusion which you have reached be acceptable to you in one role but not in another? If you can identify any conflicts, consider the following:

- Can any conflicts be resolved by further reference to the central tenets?
- Can any conflicts be resolved by revisiting the evidence and subsequent harm: benefit analysis for stakeholders?

Sometimes conflicts simply cannot be resolved, and that should be acknowledged. If a conflict has occurred, note it down in the box below, along with whether it was resolved and how. If no conflict occurred, write 'none' in this box.

CONFLICTS

8 Final decision:

In the first box below, note down the final decision which you have reached having worked your way through steps 1-7 of the framework.

In the second box, record whether there is any further work which needs to be done to confirm that decision, e.g. if there was a lack of evidence, make a suggestion for commissioning appropriate research or a plan to follow up on further evidence published in future.

In the third box, note any issues with regulation compliance. There may be occasions on which the preliminary conclusion is not compliant with current regulation / legislation and when - having reassessed both the analysis and the preliminary conclusion – the users of the framework still believe that their conclusion is correct and that current regulation / legislation needs reviewing. If this occurs it should be stated.

FINAL DECISION (your answer to the question which you have been considering)

FURTHER WORK TO BE DONE

REGULATION COMPLIANCE

Questionnaire

On the next page, you will find the questionnaire. Please fill this out AFTER you have completed the framework survey. It is important that you fill out this questionnaire as this is the information we require to modify the framework into a more useable tool. If you have any queries or concerns please email bbrown20@rvc.ac.uk or mcampbell@rvc.ac.uk.

SECTION 1: PLEASE PROVIDE SOME INFORMATION ABOUT YOURSELF BY ANSWERING THE FOLLOWING QUESTIONS:

1. How long have you been involved with the equine industry? *(please place an x by your answer)*

- Under 5 years
- 5-10 years
- 10-15 years
- 15+ years

2. What is your main role within equine industry? *(please place an x by your answer):*

- Rider/jockey/driver (competitor)
- Trainer/coach
- Owner
- Groom
- Vet
- Ambassador
- Regulator
- Sponsor
- Competition organiser
- Young rider
- Animal welfare organisation
- Academia/education/research
- Breeder

3. Is this role your main source of income? *(please place an x by your answer)*

- Yes
- No

4. In which discipline do you perform your main role as you have identified it in Q2? *(please place an x by your answer; if your role is not discipline specific, please place an x next to all that apply)*

- Dressage
- Show jumping
- Eventing
- Para dressage
- Para driving
- Flat racing
- Jump racing
- Endurance
- Polo
- Vaulting
- Driving
- Reining

5. How old are you? *(please write your answer below this question)*

………………. years old

6. Do you identify as *(please place an x by your answer)*

- Male
- Female
- Other
- Prefer not to say

SECTION 2: THE QUESTIONS IN THIS SECTION RELATE TO YOUR TRIAL USE OF THE ETHICAL FRAMEWORK FOR THE USE OF HORSES IN COMPETITIVE SPORT. PLEASE ANSWER EACH QUESTION BY PLACING AN X IN THE BOX UNDERNEATH THE PHRASE THAT YOU MOST AGREE WITH (STRONGLY DISAGREE TO STRONGLY AGREE). THERE IS A COMMENT BOX. PLEASE USE THE COMMENT BOX TO FILL IN ANY COMMENTS WHICH YOU HAVE RELATING TO THE QUESTION / WHICH EXPLAIN WHY YOU CHOSE THE PHRASE WHICH YOU DID.

7. 'I understood how to complete each part of the framework'

Strongly disagree	Mostly disagree	Somewhat disagree	Neither agree nor disagree	Somewhat agree	Mostly agree	Strongly agree

COMMENTS

8. 'I understood all the terms used in the framework'

Strongly disagree	Mostly disagree	Somewhat disagree	Neither agree nor disagree	Somewhat agree	Mostly agree	Strongly agree

COMMENTS

9. ‘The ‘worked example’ helped me understand how to use the framework’

Strongly disagree	Mostly disagree	Somewhat disagree	Neither agree nor disagree	Somewhat agree	Mostly agree	Strongly agree

COMMENTS

10. ‘The stakeholder matrix helped me to apply harm: benefit analysis to the question/issue’

Strongly disagree	Mostly disagree	Somewhat disagree	Neither agree nor disagree	Somewhat agree	Mostly agree	Strongly agree

COMMENTS

11. ‘The framework steps enabled me to come to a conclusion on the specified issue’

Strongly disagree	Mostly disagree	Somewhat disagree	Neither agree nor disagree	Somewhat agree	Mostly agree	Strongly agree

COMMENTS

12. ‘I would use this framework to make decisions in the future’

Strongly disagree	Mostly disagree	Somewhat disagree	Neither agree nor disagree	Somewhat agree	Mostly agree	Strongly agree

COMMENTS

SECTION 3: THESE QUESTIONS RELATE TO YOUR EXPERIENCE OF APPLYING THE FRAMEWORK.

13. Did you identify/ experience any conflicts (clashes of interest) while coming to a decision when using the framework? *(please place an x by your answer)*

- Yes
- No

IF YOU ANSWERED NO, PLEASE GO TO QUESTION 16

14. Was the conflict resolved? *(please place an x by your answer)*

- Yes
- No

IF YOU ANSWERED NO, PLEASE GO TO QUESTION 16

15. How did you resolve the conflict? *(please place an x by all those that apply)*

- By applying the central tenets
- By re visiting the evidence
- Other *(please specify)*

OTHER:

16. Please briefly summarise what you liked about the framework:

COMMENTS

17. Please briefly summarise what you think could be improved about the framework:

COMMENTS

Thank you for your contribution!

Appendix A

Worked Example

1 The Question is:

Should the use of performance-enhancing hind boots be allowed during Show Jumping training?

BACKGROUND

Within FEI international Show Jumping competition, the team is required to jump heights of 1.40-1.60 at a minimum speed of 350m per min. A new 'gadget' used within show jumping are the so called 'performance enhancing boots' aka 'pressure boots' 'pinch boots' 'flick boots', which can be weighted, or apply pressure and are generally made of stiffer material. The perception is that these boots alter jump stride movement and induce hyperflexion, helping the horse to clear the fence with hindlimbs 'cleanly'. Research was conducted on the use of these boots and concerns were raised that this exaggerated hyperflexion could result in an increase in falls, especially when novice horses are attempting bigger fences where balance is key. The FEI has introduced a phased banning of the use of these boots within competition, but there is no regulation regarding use during training.

2 Sport rules / laws:

FEI:
https://inside.fei.org/content/general-regs-statutes

POLO:
https://hpa-polo.co.uk/

RACING:
http://rules.britishhorseracing.com/#!/book/34

To determine whether something should be 'allowed' or 'not allowed' during competition and competition training, we first need to know what current rules, regulations, and legislation there are on this issue. Rules for each discipline are created by the regulatory body, both international and national. For example, FEI (international) covers all disciplines within this study, except racing and polo. There may be relevant rules in the general / veterinary / discipline specific regulations and there is also the FEI Code of Conduct. You can look up the rules for each discipline by clicking the relevant links on the left hand side of this page or by copying and pasting those links into your internet browser. Note that participants in Round 2 testing are being asked different questions from each other and therefore not all of the links shown will be relevant to your discipline / the particular question which you have been asked. Laws can refer to legislation like The Animal Welfare Act, 2006. You can search for legislation via Google or on a government website like gov.uk. Are there any sports rules / laws which are relevant to the question which you are answering? If so, make a brief note in the sport rules / laws box on the next page. It will be helpful to write down who made the regulation (e.g. BHA) and what it is.

SPORT RULES/LAWS (you are not expected to fill this entire box)

FEI jumping rules – phased banning of performance enhancing boots.

FEI code of conduct - at all times the welfare of the horse must be paramount. Welfare of the horse must never be subordinated to competitive or commercial influence (including training methods).

FEI Article 49 – Limb sensitivity examination with the aim to protect welfare of the horse, level playing field. Pressure test is to detect any abnormal reaction to pressure – indicates physical pain as nociceptors within the skin relay this information to the CNS which results in a pain response to move away from the ‘noxious’ stimuli.

Animal Welfare Act, 2006 - Need to be protected from pain, suffering, injury, and disease.

3 Evidence:

In order to make an ethical decision, we need to consider what evidence is available which would support a decision one way or the other. There are several types of evidence, which have varying degrees of quality. The information boxes below will help you to identify different types of evidence and how to find them. You may also already know some evidence about the question which you are considering. In the box on the next page, write down any evidence which you already know or have been able to find about the question, including who the author (organisation or person) was and a brief note on what the evidence said.

RESEARCH ARTICLE. The 'evidence' within a research article is based on the results of some form of scientific research, which is usually assessed by other scientists before it is published. Where possible, you should aim to try and include this as one of your main sources of evidence, as this is considered 'objective' – the researchers do not have a vested interest towards the outcome of the research. To search for a research article online, you can use 'Google Scholar'. If you don't already have this browser, type 'Google Scholar' into Google, click on the result and use this browser to search for information, like you would do for normal Googling. Click on one of the relevant results, this should usually take you to a 'summary' or 'abstract', a short paragraph about the research and its results. If you find relevant information, you can read these summaries and note them down in the 'evidence' box on the next page.

BOOKS, REPORTS, MAGAZINE ARTICLES. You may have read some information in a book or magazine that relates to the question or you can search for this information – in a library, Google books or Google. For magazine articles, TheHorse.com, the FEI website and Horse & Hound are good places to start. These articles are generally based on research findings or are written by an 'expert' in the area, e.g. a vet, but are usually easier to understand than research articles. It might be difficult to find something directly relevant but there may be more information on this area in other species or sports.

STAKEHOLDER EXPERT OPINION/ PERSONAL EXPERIENCE. You may have attended a conference or a seminar about the issue. You can include this within your evidence box. You may also have some personal experience which you would like to include.

EVIDENCE (there is an additional box on the next page if you require extra space)

Murphy 2008 – In this research they found that 70% of Nations cup competitors, 66.8% Grand Prix competitors, 47.3% age class competitors used these boots, with only a small number changing boots or removing them after warm up. This shows that the use of these boots was quite popular.

Murphy 2009 - Investigated effect of weighted hindlimb boots on jump stride kinematics in show jumping when jumping 1.25m oxer. Found horses consistently had greater hindlimb elevation with weighted boots. Weighted boots place additional loading on distal limb. Therefore, there may be an increase in the risk of hindlimb tissue and lumbar musculature damage. The boots may also impact the horse's comfort.

Wickler et al, 2004 - found that weighting the hind pasterns increased swing duration, but stance phase decrease was related to speed rather than weights. Also found an increase in 'metabolic rate', an increase in oxygen consumption – horse has to work harder.

Clayton et al., 2011 -weight added to distal limb = need increased force to pull limb off ground into swing phase. This means there is an increased need for 'positive' work in hip and tarsal extensors and an increased need in 'negative' work is needed across stifle and MTP joints. These increased needs *might* impact upon soundness.

Clayton & Van Weeren, 2012. Show Jumping places extreme loading stress on the hindlimbs during lift off and the forelimbs during landing.

Horse & Hound – Murphy wrote an article in Horse & Hound expressing his concern as these boots may cause an increase in falls and injuries for horses because of hind end hyperflexion.

EVIDENCE CONTINUED (you are not required to fill this box; it is here if you need extra space)

4 Stakeholder harm: benefit analysis

In order to come to a decision on whether something is ‘right or wrong’, a common approach is based on ‘utilitarianism’, which looks at the consequences of an action. This is where an action or decision is considered ethically ‘right’ if it achieves the greatest good (or benefit) for the greatest number. Any decision will affect many people through knock on consequences, and so it is important to weigh up the ‘harm’ and ‘benefit’ for each stakeholder affected by the decision. Below are examples of ‘equine’ (red) ‘human’ (blue) and ‘other’ (purple) stakeholders, that may be impacted by the question under consideration. Consider which stakeholders are likely to be impacted and in the table overleaf (the stakeholder matrix) note down the stakeholder, and the potential ‘harm’ and ‘benefit’ to that stakeholder, if the answer to the question is ‘yes’. Not all stakeholders are relevant in every situation and there may be stakeholders that you identify as impacted that are not listed here. As the goal of this framework it to come to an ethical decision in relation to the *use of horses* in competitive sport, the stakeholder matrix ***MUST include at least one equine stakeholder.***

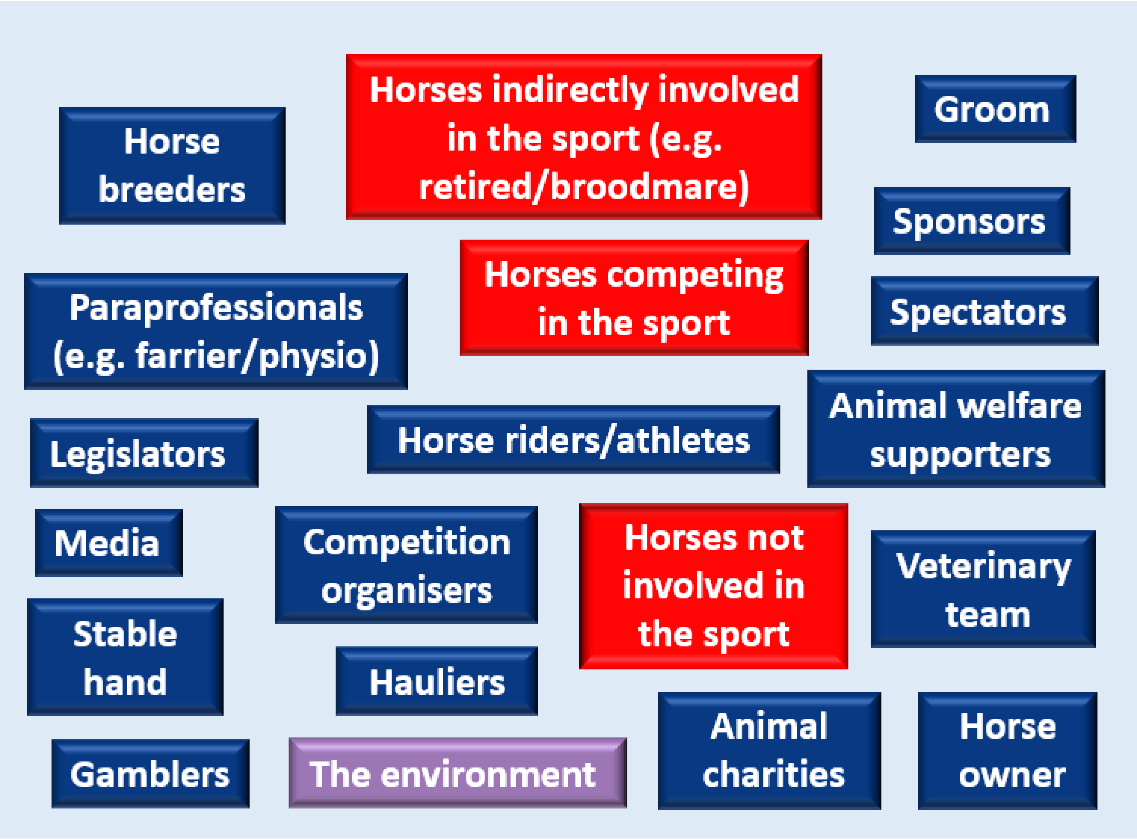

Stakeholder	Potential harm to stakeholder	Potential benefit to stakeholder
**Horses competing in the sport**	Pain<br>Possible long term damage to back/limbs<br>Increased chance of injury<br>Jumping is less ‘attractive’ for the horse<br>Increased chance of fatigue	
**Horse Rider**	Horse could retire earlier due to injury and/or have lots of time off work.	Increased chance of winning
**Horse Owner**	Injured horse	Increased winnings
**Spectators**	May see an increase in injuries which could reduce the ‘social licence’ of horses Show Jumping.	More of a ‘spectacle’
**Groom**	May find it unsettling to see horse in discomfort / injured.<br>Increased workload looking after injured horse	Increased chance of being part of the ‘winning team’

5 Your preliminary decision:

In the box below, note down your preliminary answer to the question which you are considering, based on the harm: benefit analysis of stakeholders' interests.

YOUR PRELIMINARY DECISION

The harm:benefit analysis suggests that there are multiple benefits to many human stakeholders of allowing the use of the boots during training. However, there is also some evidence of the use of the boots causing actual and potential harms to horses. When the stakeholder matrix is looked at over all, there seem to be more benefits to humans than harms to horses listed. Therefore following the aim of 'maximising the benefit to the most', a preliminary decision would be made in favour of allowing the use of the boots in training.

6 The Central Tenets:

Your preliminary decision should be tested against the ‘central tenets’ of the framework which are listed in the box to the right. This assists ‘weighting’ different stakeholder interests if the harm: benefit analysis suggests that a particular decision is of benefit to one stakeholder and to the detriment of another. In other words, applying the central tenets to your preliminary decision helps you decide whose interests should be given precedence. For example, suppose that you have gathered the evidence and applied the harm: benefit analysis and made an initial decision in favour of an action which is economically beneficial to humans but also carries an identifiable risk of an avoidable harm to horses. When you check your initial decision against the central tenets, you will see that one of the tenets states that there must be ‘identification and mitigation against avoidable, unnecessary risk to horses’. Your initial decision to allow human economic benefits to override equine harms is in contradiction to this tenet and therefore your initial conclusion / decision needs to be reconsidered.

The Central Tenets

Minimisation of negative welfare effects and maximisation of positive welfare effects for horses.

Identification of and mitigation against avoidable, unnecessary risk to horses.

Compliance with governing body regulations and the law.

7 Acknowledging and resolving conflicts:

At the moment, you are testing this framework individually but in Round 3 of testing and thereafter it will normally be used by groups of people working together to consider an ethical issue. Conflicts may occur between the interests of those who are together using the framework to consider a question. Such conflicts may make it difficult for all involved to subscribe to the decision which has been reached by applying this framework. Consider whether, even when you are testing the framework individually, you can identify such conflicts. For example, if you play more than one role in your discipline (perhaps you are both a rider and a breeder or owner) would the conclusion which you have reached be acceptable to you in one role but not in another? If you can identify any conflicts, consider the following:

- ❖ Can any conflicts be resolved by further reference to the central tenets?
- ❖ Can any conflicts be resolved by revisiting the evidence and subsequent harm: benefit analysis for stakeholders?

Sometimes conflicts simply cannot be resolved, and that should be acknowledged. If a conflict has occurred, note it down in the box below, along with whether it was resolved and how. If no conflict occurred, write 'none' in this box.

CONFLICTS

None

8 Final decision:

In the first box below, note down the final decision which you have reached having worked your way through steps 1-7 of the framework.

In the second box, record whether there is any further work which needs to be done to confirm that decision e.g. if there was a lack of evidence, make a suggestion for commissioning appropriate research or a plan to follow up on further evidence published in future.

In the third box, note any issues with regulation compliance. There may be occasions on which the preliminary conclusion is not compliant with current regulation / legislation and when - having reassessed both the analysis and the preliminary conclusion – the users of the framework still believe that their conclusion is correct and that current regulation / legislation needs reviewing. If this occurs it should be stated.

FINAL DECISION (your answer to the question which you have been considering)

The preliminary decision was to allow the use of the boots in training because it was felt that there were more benefits to humans to doing so than there were equine harms. However, when that decision is tested against the central tenets we can see that it contravenes the tenet :'Minimisation of negative welfare effects and maximisation of positive welfare effects for horses', since there is some evidence (listed in the 'evidence box') that the boots cause discomfort and injury, and these negative welfare effects could be removed by not allowing the use of the boots. The preliminary decision therefore has to be reconsidered in light of the application of the central tenets, and the final decision is NOT to allow the use of boots in training, as this best protects equine welfare. However, there is a lack of evidence on the subject, and this decision may itself need to be reviewed as further evidence becomes available – see 'Further work to be done' below.

FURTHER WORK TO BE DONE

Further work is needed to determine whether the changes in locomotion which have been shown to result from use of the boots do actually impact injury rates and types.

Could consider commissioning research to address this deficit in knowledge.

Certainly review new literature every 4 months and adapt the decision in light of new evidence as it becomes available.

REGULATION COMPLIANCE

Currently no regulation about the use of boots in training.
