# Supplementary material for: Testing and Refining the Ethical Framework for the Use of Horses in Sport
Source: Animals (Basel). 2023 May 31;13(11):1821. doi: 10.3390/ani13111821 (PMC10252045; doi:10.3390/ani13111821)
Supplement: Supplementary file 1 [file animals-13-01821-s001.zip › Document S5 Round 3 ethical framework application document.pdf]

Development of an ethical framework tool for the use of horses in competitive sport

'Round 3' framework survey

Framework Process

Step 1.

What is the question and background?

Step 2.

Identify any sport rules (regulations) or laws related to the question

Step 3.

Identify evidence. You can include:

Research   Books/magazines   Expert opinion   Personal experience

Step 4

Carry out stakeholder harm: benefit analysis

Step 5

Think about the 'key considerations'

Step 6

Come to a decision on the question

If you experience conflict(s) within your group whilst reaching a decision, note that down

Can this conflict be resolved by reexamining the evidence and harm: benefit analysis?

Note down any further work (research) which you think needs doing to help answer the question

1 The Question is:

Should a novice horse be allowed to complete a maximum distance of (280 miles; 450 km) in their first competition year?

Background

Under Endurance GB rules, a novice horse can compete in up to ten endurance competitions to a maximum distance of 450km (280 miles) in their first endurance season.

2 Sport rules / laws:

FEI:
https://inside.fei.org/content/general-regs-statutes

POLO:
https://hpa-polo.co.uk/

RACING:
http://rules.britishhorseracing.com/#!/book/34

To determine whether something should be 'allowed' or 'not allowed' during competition and competition training, we first need to know what current rules, regulations, and legislation there are on this issue. Rules for each discipline are created by the regulatory body, both international and national. For example, FEI (international) covers all disciplines within this study, except racing and polo. There may be relevant rules in the general / veterinary / discipline specific regulations and there is also the FEI Code of Conduct. You can look up the rules for each discipline by clicking the relevant links on the left hand side of this page or by copying and pasting those links into your internet browser. Note that participants in Round 3 testing are being asked different questions from each other and therefore not all of the links shown will be relevant to your discipline / the particular question which you have been asked. Laws can refer to legislation like The Animal Welfare Act, 2006. You can search for legislation via Google or on a government website like gov.uk. Are there any sports rules / laws which are relevant to the question which you are answering? If so, make a brief note in the sport rules / laws box on the next page. It will be helpful to write down who made the regulation (e.g. BHA) and what it is.

SPORT RULES/LAWS (you are not expected to fill this entire box)

3 Evidence:

In order to make an ethical decision, we need to consider what evidence is available which would support a decision one way or the other. There are several types of evidence, which have varying degrees of quality. The information boxes below will help you to identify different types of evidence and how to find them. You may also already know some evidence about the question which you are considering. In the box on the next page, write down any evidence which you already know or have been able to find about the question, including who the author (organisation or person) was and a brief note on what the evidence said.

RESEARCH ARTICLE. The 'evidence' within a research article is based on the results of some form of scientific research, which is usually assessed by other scientists before it is published. Where possible, you should aim to try and include this as one of your main sources of evidence, as this is considered 'objective' – the researchers do not have a vested interest towards the outcome of the research. To search for a research article online, you can use 'Google Scholar'. If you don't already have this browser, type 'Google Scholar' into Google, click on the result and use this browser to search for information, like you would do for normal Googling. Click on one of the relevant results, this should usually take you to a 'summary' or 'abstract', a short paragraph about the research and its results. If you find relevant information, you can read these summaries and note them down in the 'evidence' box on the next page.

BOOKS, REPORTS, MAGAZINE ARTICLES. You may have read some information in a book or magazine that relates to the question or you can search for this information – in a library, Google books or Google. For magazine articles, TheHorse.com, the FEI website and Horse & Hound are good places to start. These articles are generally based on research findings or are written by an 'expert' in the area, e.g. a vet, but are usually easier to understand than research articles. It might be difficult to find something directly relevant but there may be more information on this area in other species or sports.

STAKEHOLDER EXPERT OPINION/ PERSONAL EXPERIENCE. You may have attended a conference or a seminar about the issue. You can include this within your evidence box. You may also have some personal experience which you would like to include.

EVIDENCE (there is an additional box on the next page if you require extra space)

EVIDENCE (you do not have to fill in this box, it is here if you require extra space.)

EVIDENCE (you do not have to fill in this box, it is here if you require extra space.)

4 Stakeholder harm: benefit analysis

In order to come to a decision on whether something is 'right or wrong', a common approach is based on 'utilitarianism', which looks at the consequences of an action. This is where an action or decision is considered ethically 'right' if it achieves the greatest good (or benefit) for the greatest number. Any decision will affect many people through knock on consequences, and so it is important to weigh up the 'harm' and 'benefit' for each 'stakeholder'.

A 'stakeholder' is anyone affected by the decision and may be impacted by the question under consideration. In the table on the next page (the stakeholder matrix) consider which stakeholders are likely to be impacted and write down the potential 'harm' and 'benefit' to that stakeholder, if the answer to the question which you are considering were 'yes'. Not all stakeholders are relevant in every situation, you can leave those boxes blank. There may be stakeholders that you identify as impacted that are not listed here and you can include them in the table if you wish to do so (there is blank space on the last page of the table). As the goal of this framework it to come to an ethical decision in relation to the *use of horses* in competitive sport, the stakeholder matrix ***MUST include at least one equine stakeholder.***

Stakeholder	Potential harm to stakeholder	Potential benefit to stakeholder
Horses competing in the sport		
Horses indirectly involved in the sport (e.g. retired/ broodmare)		
Horses not involved in the sport		
Horse breeders		
Horse riders/athletes		

Stakeholder	Potential harm to stakeholder	Potential benefit to stakeholder
Animal welfare supporters		
Horse owner		
Legislators		
Stable hand		
Veterinary team		

Stakeholder	Potential harm to stakeholder	Potential benefit to stakeholder
Paraprofessionals (e.g. farrier, physio)		
Competition organisers		
Animal charities		
Groom		
Spectator		

Stakeholder	Potential harm to stakeholder	Potential benefit to stakeholder
Media		
Gamblers		
The environment		
Hauliers		

Stakeholder	Potential harm to stakeholder	Potential benefit to stakeholder

5 Key Considerations:

When coming to a decision, it is important to include the **'key considerations'** of the framework, which are listed in the box below. This assists 'weighting' different stakeholder interests if the harm: benefit analysis suggests that a particular decision is of benefit to one stakeholder and to the harm of another. In other words, including the 'key considerations' within your decision helps you decide whose interests should be given priority. This is to ensure that human benefits do not override potential risks to horses.

Key Considerations

- **Minimisation of negative welfare and maximisation of positive welfare for horses.**
- **Identification of and prevention against avoidable, unnecessary risk to horses.**
- **Compliance with governing body regulations and the law.**

6 Your decision:

In the first box on the next page, note down your decision, based on the evidence, the harm: benefit analysis and the key considerations. It is important to remember that it is not always possible to come to a straight yes or no answer. You may come to a decision that has caveats, or you may need further evidence to come to a strong decision; this is completely acceptable.

In the second box, record whether there is any further work which needs to be done to confirm that decision, e.g. if there was a lack of evidence, make a suggestion for commissioning appropriate research or a plan to follow up on further evidence published in future.

In the third box, note any issues with regulation compliance. There may be occasions when your decision is not compliant with current regulation / legislation and when - having reassessed both the analysis and the decision – the users of the framework still believe that their conclusion is correct and that current regulation / legislation needs reviewing. If this occurs it should be stated.

IMPORTANT: When using the framework as a group, disagreements (conflicts) on the decision can occur. These conflicts need to be noted down and you should then attempt to see if you can reach an agreement by using the process described on page 21 of the framework and in the example on page 21.

FINAL DECISION (your answer to the question which you have been considering)

FINAL DECISION (you do not have to fill this box, it is here if you require extra space)

FURTHER WORK TO BE DONE

REGULATION COMPLIANCE

Acknowledging and resolving conflicts:

Conflicts may occur between the interests of people who are using the framework together to consider a question. Such conflicts may make it difficult for all involved to agree with the decision which is being reached. If any conflict has occured consider the following:

- Can any conflicts be resolved by further reference to the key considerations?
- Can any conflicts be resolved by revisiting the evidence and harm: benefit analysis for stakeholders?

Sometimes conflicts simply cannot be resolved, and that should be acknowledged. If a conflict has occurred, note it down in the box below, along with whether it was resolved and how.

CONFLICTS
