# Supplementary material for: Testing and Refining the Ethical Framework for the Use of Horses in Sport
Source: Animals (Basel). 2023 May 31;13(11):1821. doi: 10.3390/ani13111821 (PMC10252045; doi:10.3390/ani13111821)
Supplement: Supplementary file 1 [file animals-13-01821-s001.zip › Document S6 Round 3 worked example.pdf]

Development of an ethical framework tool for the use of horses in competitive sport

'Round 3' framework survey

Framework Process

Step 1.

What is the question and background?

Step 2.

Identify any sport rules (regulations) or laws related to the question

Step 3.

Identify evidence. You can include:

Research   Books/magazines   Expert opinion   Personal experience

Step 4

Carry out stakeholder harm: benefit analysis

Step 5

Think about the 'key considerations'

Step 6

Come to a decision on the question

If you experience conflict(s) within your group whilst reaching a decision, note that down

Can this conflict be resolved by reexamining the evidence and harm: benefit analysis?

Note down any further work (research) which you think needs doing to help answer the question

1 The Question is:

Should the use of performance-enhancing hind boots be allowed during Showjumping training?

Background

Within FEI international Showjumping competition, the team is required to jump heights of 1.40-1.60 at a minimum speed of 350m per min. A new ‘gadget’ used within showjumping are the so called ‘performance enhancing boots’ aka ‘pressure boots’ ‘pinch boots’ ‘flick boots’, which can be weighted, or apply pressure and are generally made of stiffer material. The perception is that these boots alter jump stride movement and induce hyperflexion, helping the horse to clear the fence with hindlimbs ‘cleanly’. Research was conducted on the use of these boots and concerns were raised that this exaggerated hyperflexion could result in an increase in falls, especially when novice horses are attempting bigger fences where balance is key. The FEI has introduced a phased banning of the use of these boots within competition, but there is no regulation regarding use during training.

2 Sport rules / laws:

FEI:
https://inside.fei.org/content/general-regs-statutes

POLO:
https://hpa-polo.co.uk/

RACING:
http://rules.britishhorseracing.com/#!/book/34

To determine whether something should be 'allowed' or 'not allowed' during competition and competition training, we first need to know what current rules, regulations, and legislation there are on this issue. Rules for each discipline are created by the regulatory body, both international and national. For example, FEI (international) covers all disciplines within this study, except racing and polo. There may be relevant rules in the general / veterinary / discipline specific regulations and there is also the FEI Code of Conduct. You can look up the rules for each discipline by clicking the relevant links on the left hand side of this page or by copying and pasting those links into your internet browser. Note that participants in Round 3 testing are being asked different questions from each other and therefore not all of the links shown will be relevant to your discipline / the particular question which you have been asked. Laws can refer to legislation like The Animal Welfare Act, 2006. You can search for legislation via Google or on a government website like gov.uk. Are there any sports rules / laws which are relevant to the question which you are answering? If so, make a brief note in the sport rules / laws box on the next page. It will be helpful to write down who made the regulation (e.g. BHA) and what it is.

SPORT RULES/LAWS (you are not expected to fill this entire box)

FEI jumping rules – phased banning of performance enhancing boots.

FEI code of conduct - at all times the welfare of the horse must be paramount. Welfare of the horse must never be subordinated to competitive or commercial influence (including training methods).

FEI Article 49 – Limb sensitivity examination with the aim to protect welfare of the horse, level playing field. Pressure test is to detect any abnormal reaction to pressure – indicates physical pain as nociceptors within the skin relay this information to the CNS which results in a pain response to move away from the ‘noxious’ stimuli.

Animal Welfare Act, 2006 - Need to be protected from pain, suffering, injury, and disease.

3 Evidence:

In order to make an ethical decision, we need to consider what evidence is available which would support a decision one way or the other. There are several types of evidence, which have varying degrees of quality. The information boxes below will help you to identify different types of evidence and how to find them. You may also already know some evidence about the question which you are considering. In the box on the next page, write down any evidence which you already know or have been able to find about the question, including who the author (organisation or person) was and a brief note on what the evidence said.

RESEARCH ARTICLE. The 'evidence' within a research article is based on the results of some form of scientific research, which is usually assessed by other scientists before it is published. Where possible, you should aim to try and include this as one of your main sources of evidence, as this is considered 'objective' – the researchers do not have a vested interest towards the outcome of the research. To search for a research article online, you can use 'Google Scholar'. If you don't already have this browser, type 'Google Scholar' into Google, click on the result and use this browser to search for information, like you would do for normal Googling. Click on one of the relevant results, this should usually take you to a 'summary' or 'abstract', a short paragraph about the research and its results. If you find relevant information, you can read these summaries and note them down in the 'evidence' box on the next page.

BOOKS, REPORTS, MAGAZINE ARTICLES. You may have read some information in a book or magazine that relates to the question or you can search for this information – in a library, Google books or Google. For magazine articles, TheHorse.com, the FEI website and Horse & Hound are good places to start. These articles are generally based on research findings or are written by an 'expert' in the area, e.g. a vet, but are usually easier to understand than research articles. It might be difficult to find something directly relevant but there may be more information on this area in other species or sports.

STAKEHOLDER EXPERT OPINION/ PERSONAL EXPERIENCE. You may have attended a conference or a seminar about the issue. You can include this within your evidence box. You may also have some personal experience which you would like to include.

EVIDENCE (there is an additional box on the next page if you require extra space)

Murphy 2008 – In this research they found that 70% of Nations cup competitors, 66.8% Grand Prix competitors, 47.3% age class competitors used these boots, with only a small number changing boots or removing them after warm up. This shows that the use of these boots was quite popular.

Murphy 2009 - Investigated effect of weighted hindlimb boots on jump stride kinematics in show jumping when jumping 1.25m oxer. Found horses consistently had greater hindlimb elevation with weighted boots. Weighted boots place additional loading on distal limb. Therefore, there may be an increase in the risk of hindlimb tissue and lumbar musculature damage. The boots may also impact the horse's comfort.

Wickler et al, 2004 - found that weighting the hind pasterns increased swing duration, but stance phase decrease was related to speed rather than weights. Also found an increase in 'metabolic rate', an increase in oxygen consumption – horse has to work harder.

Clayton et al., 2011 -weight added to distal limb = need increased force to pull limb off ground into swing phase. This means there is an increased need for 'positive' work in hip and tarsal extensors and an increased need in 'negative' work is needed across stifle and MTP joints. These increased needs *might* impact upon soundness.

Clayton & Van Weeren, 2012. Showjumping places extreme loading stress on the hindlimbs during lift off and the forelimbs during landing.

Horse & Hound – Murphy wrote an article in Horse & Hound expressing his concern as these boots may cause an increase in falls and injuries for horses because of hind end hyperflexion.

EVIDENCE (you do not have to fill in this box, it is here if you require extra space.)

EVIDENCE (you do not have to fill in this box, it is here if you require extra space.)

4 Stakeholder harm: benefit analysis

In order to come to a decision on whether something is 'right or wrong', a common approach is based on 'utilitarianism', which looks at the consequences of an action. This is where an action or decision is considered ethically 'right' if it achieves the greatest good (or benefit) for the greatest number. Any decision will affect many people through knock on consequences, and so it is important to weigh up the 'harm' and 'benefit' for each 'stakeholder'.

A 'stakeholder' is anyone affected by the decision and may be impacted by the question under consideration. In the table on the next page (the stakeholder matrix) consider which stakeholders are likely to be impacted and write down the potential 'harm' and 'benefit' to that stakeholder, if the answer to the question which you are considering were 'yes'. Not all stakeholders are relevant in every situation, you can leave those boxes blank. There may be stakeholders that you identify as impacted that are not listed here and you can include them in the table if you wish to do so (there is blank space on the last page of the table). As the goal of this framework it to come to an ethical decision in relation to the *use of horses* in competitive sport, the stakeholder matrix ***MUST include at least one equine stakeholder.***

Stakeholder	Potential harm to stakeholder	Potential benefit to stakeholder
Horses competing in the sport	Pain<br>Possible long term damage to back/limbs<br>Increased chance of injury<br>Jumping is less 'attractive' for the horse<br>Increased chance of fatigue	
Horses indirectly involved in the sport (e.g. retired/ broodmare)		
Horses not involved in the sport		
Horse breeders		
Horse riders/athletes	Horse could retire earlier due to injury and/or have lots of time off work.	Increased chance of winning

Stakeholder	Potential harm to stakeholder	Potential benefit to stakeholder
Animal welfare supporters		
Horse owner	Injured horse	Increased winnings
Legislators		
Stable hand		
Veterinary team		

Stakeholder	Potential harm to stakeholder	Potential benefit to stakeholder
Paraprofessionals (e.g. farrier, physio)		
Competition organisers		
Animal charities		
Groom	May find it unsettling to see horse in discomfort / injured. Increased workload looking after injured horse	Increased chance of being part of the ‘winning team’.
Spectator	May see an increase in injuries which could reduce the ‘social licence’ of horses showjumping.	More of a ‘spectacle’

Stakeholder	Potential harm to stakeholder	Potential benefit to stakeholder
Media		
Gamblers		
The environment		
Hauliers		

Stakeholder	Potential harm to stakeholder	Potential benefit to stakeholder

5 Key Considerations:

When coming to a decision, it is important to include the **'key considerations'** of the framework, which are listed in the box below. This assists 'weighting' different stakeholder interests if the harm: benefit analysis suggests that a particular decision is of benefit to one stakeholder and to the harm of another. In other words, including the 'key considerations' within your decision helps you decide whose interests should be given priority. This is to ensure that human benefits do not override potential risks to horses.

Key Considerations

- **Minimisation of negative welfare and maximisation of positive welfare for horses.**
- **Identification of and prevention against avoidable, unnecessary risk to horses.**
- **Compliance with governing body regulations and the law.**

6 Your decision:

In the first box on the next page, note down the decision, based on the evidence, the harm: benefit analysis and the key considerations. It is important to remember that it is not always possible to come to a straight yes or no answer. You may come to a decision that has caveats, or you may need further evidence to come to a strong decision; this is completely acceptable.

In the second box, record whether there is any further work which needs to be done to confirm that decision, e.g. if there was a lack of evidence, make a suggestion for commissioning appropriate research or a plan to follow up on further evidence published in future.

In the third box, note any issues with regulation compliance. There may be occasions when your decision is not compliant with current regulation / legislation and when - having reassessed both the analysis and the decision – the users of the framework still believe that their conclusion is correct and that current regulation / legislation needs reviewing. If this occurs it should be stated.

IMPORTANT: When using the framework as a group, disagreements (conflicts) on the decision can occur. These conflicts need to be noted down and you should then attempt to see if you can reach an agreement by using the process described on page 21 of the framework.

FINAL DECISION (your answer to the question which you have been considering)

The harm:benefit analysis suggests that there are multiple benefits to many human stakeholders by allowing the use of the boots during training. However, there is also some evidence of the use of the boots causing actual and potential harms to horses. When we include the key considerations in the decision, allowing the use of these performance enhancing hind boots boots during training goes against 'Minimisation of negative welfare and maximisation of positive welfare for horses.' This is because there is some evidence (listed in the 'evidence box') that the boots cause discomfort and injury, and this negative welfare could be removed by not allowing the use of the boots. However, there is a lack of evidence on the subject, and this decision may itself need to be reviewed as further evidence becomes available – see 'Further work to be done' below.

FINAL DECISION (you do not have to fill this box, it is here if you require extra space)

FURTHER WORK TO BE DONE

Further work is needed to determine whether the changes in locomotion which have been shown to result from use of the boots do actually impact injury rates and types.

Could consider commissioning research to address this deficit in knowledge.

Certainly review new literature every 4 months and adapt the decision in light of new evidence as it becomes available.

REGULATION COMPLIANCE

Currently no regulation about the use of boots in training.

Acknowledging and resolving conflicts:

Conflicts may occur between the interests of people who are using the framework together to consider a question. Such conflicts may make it difficult for all involved to agree with the decision which is being reached by applying this framework. If any conflict has occured consider the following:

- Can any conflicts be resolved by further reference to the key considerations?
- Can any conflicts be resolved by revisiting the evidence and harm: benefit analysis for stakeholders?

Sometimes conflicts simply cannot be resolved, and that should be acknowledged. If a conflict has occurred, note it down in the box below, along with whether it was resolved and how.

CONFLICTS
