# Supplementary material for: Testing and Refining the Ethical Framework for the Use of Horses in Sport
Source: Animals (Basel). 2023 May 31;13(11):1821. doi: 10.3390/ani13111821 (PMC10252045; doi:10.3390/ani13111821)
Supplement: Supplementary file 1 [file animals-13-01821-s001.zip › Table S1 Predetermined ethical questions for participants used in each round per discipline.pdf]

Discipline	Question
Round 1	
**All**	Should omeprazole be permitted for use in horses during competition?
Round 2	
**Eventing**	Should young horse classes be allowed?
**Reining**	
**Dressage**	
**Showjumping**	
**Para dressage**	Should anyone other than the athlete/ competitor be allowed to ride / train / school horses competing at all grades in para dressage competitions after arrival at the competition venue?
**Endurance**	Should there be a maximum speed restriction during national (wherever held) and international endurance rides?
**Carriage driving**	Should weight limits for horses and ponies in carriage driving competitions take account of the combined weight of the carriage, driver and backstepper, rather than just the carriage?
**Para Carriage**	
**Flat racing**	Should racing of 2 year olds be allowed?
**Jump racing**	Should the number of runners in The Grand National be further reduced?
**Polo**	Should there be an upper weight limit for polo players?
Round 3	
**Eventing**	Should Young Horse Classes be allowed?
**Showjumping**	
**Dressage**	Should the use of bitless bridles during competition be allowed?
**Para dressage**	
**Mixed**	
**Endurance**	Should a novice horse be allowed to compete a maximum distance of (280 miles; 450 km) in their first competition year?
**Carriage driving**	Should weight limits for horses and ponies in carriage driving competitions take account of the combined weight of the carriage, driver and backstepper, rather than just the carriage?
**Flat racing**	Should racing of 2 year olds be allowed?
**Jump racing**	Should the number of runners in The Grand National be further reduced?
**Polo**	Should there be an upper weight limit for polo players?
